# Global COVID-19 vaccine acceptance level and its determinants: an umbrella review

**DOI:** 10.1186/s12889-023-17497-4

**Published:** 2024-01-02

**Authors:** Biruk Beletew Abate, Befkad Derese Tilahun, Berihun Mulu Yayeh

**Affiliations:** 1 Pediatrics and Child Health Nursing, College of Health Science, Woldia University, Woldia, Ethiopia; 2 Department of Nursing, College of Health Science, Woldia University, Woldia, Ethiopia; 3 School of Public Health, College of Health Sciences, Woldia University, Woldia, Ethiopia

**Keywords:** COVID-19, Vaccine acceptance, Umbrella review

## Abstract

**Background:**

The COVID-19 vaccination is essential for reducing disease burden on a worldwide scale. The success of this strategy will largely depend on how well vaccines are received. Previous reviews had produced contradictory results, and there had been no umbrella review. Therefore, the objective of this umbrella review was to combine the contradictory data regarding the COVID-19 vaccination’s global acceptance rate and its contributing factors.

**Methods:**

Using PRISMA guideline, PubMed, Embase, Scopus, Web of Sciences, Cochrane Database of Systematic Reviews, Scopus and Google Scholar which reported COVID-19 vaccine acceptance and/or its determinants were searched. The quality of the included studies was assessed using Assessment of Multiple Systematic Reviews (AMSTAR). A weighted inverse variance random-effects model was applied to find the pooled estimates. The subgroup analysis, heterogeneity, publication bias and sensitivity analysis were also assessed.

**Result:**

Twenty-two SRM with 10,433,306 study participants were included. The pooled COVID-19 vaccine acceptance rate globally is found to be 60.23 (95% CI: 58.27, 62.18). In low-income countries, the pooled level of COVID-19 vaccine acceptance was found to be 54.07(50.31, 57.83) while this magnitude is 64.32 (62.24,66.40) among studies across the globe. Higher level of education (AOR =1.96; 95% CI:1.20, 2.73), good level of knowledge (2.20; 95% CI:1.36, 3.03), favourable attitude (AOR =4.50; 95% CI:2.89, 6.12), previous history of COVID-19 infection (AOR =3.41; 95% CI:1.77, 5.06), male sex (AOR =1.62; 95% CI:1.47, 1.77), and chronic disease (AOR =1.54; 95% CI:1.18, 1.90) were predictors of COVID-19 vaccine acceptance.

**Conclusion:**

The pooled level of COVID-19 vaccine acceptance highly varied and found to be unacceptably low particularly in low-income countries. Higher level of education, good level of knowledge, favourable attitude, previous history of COVID-19, male sex, and chronic disease were factors of COVID-19 vaccine acceptance rate. A collaborative effort of stakeholders such as policymakers, and vaccine campaign program planners is needed to improve the acceptance rate of COVID-19 vaccine.

**Supplementary Information:**

The online version contains supplementary material available at 10.1186/s12889-023-17497-4.

## Introduction

Coronavirus disease (COVID-19) is an infectious disease caused by the SARS-CoV-2 (severe acute respiratory syndrome coronavirus 2) virus. The virus that causes COVID-19, was declared a public health emergency of international concern on 30 January 2020 and a pandemic on 11 March 2020 [1]. Worldwide, more than 1.2 million new cases and more than 7100 deaths were reported in the last 28 days (22 May to 18 June 2023) [2]. Although Reported cases are not an accurate representation of infection rates due to the reductions in testing and reporting globally, as of 18 June 2023, over 768 million confirmed cases and over 6.9 million deaths have been reported globally [2]. Globally, novel coronavirus illness (COVID-19) has emerged as a severe public health issue. COVID-19 infected around 768 million people, and more than 6.9 million people died as a result [1]. In order to effectively combat communicable diseases, three basic preventative measures must be taken: to decrease the reservoir of infection, to protect the susceptible host, and to obstruct the mechanism of transmission. It is well acknowledged that the COVID-19, a disease that mostly spreads through the air, is out of control in many countries. Many states implemented the WHO’s recommended precautions, including as social and physical distancing, masking, hygienic practices, isolation of the ill, and quarantining cases of potential exposure [2]. These measures by themselves are insufficient to contain the pandemic crisis. The COVID-19 vaccine is the only thing regarded as a reliable long-term remedy as a result. Widespread vaccine hesitancy, however, can impede vaccine uptake and effectiveness since it involves a number of attitudes and beliefs about immunization that result in postponing or outright refusing vaccination [3, 4].

According to Fisk, two categories of obstacles that prevent vaccination uptake: structural and attitudinal. Systematic problems called structural barriers make it difficult for a person to obtain services. They include elements that affect accessibility and affordability, like price, store location, or transportation. Attitudinal obstacles are views or ideas that affect a person’s desire to seek out and accept a service if they are at risk. Concerns and lack of trust in the government and the healthcare system, attitudes towards communicable diseases, attitudes towards immunizations, and contentment with the quality of the treatments obtained are a few of them. Among all attitudinal barriers, public trust is particularly important for deciding vaccination status [5].

There has been a little increase in deaths but a slight drop in cases in the African region. 10.8 million COVID-19 cases were present on the continent as of 24 February 2023, with 228,738 deaths (CFR: 2.1%), and 9.8 million recoveries (93.8%). Africa accounted for 1.3% of cases (757.2 million) and 1.2% of fatalities (6.8 million) reported globally. The WHO African Region (WHO AFR) was responsible for 76.2% of the deaths (174,191) and 82.7% of the cases (8.9 million). As of 24 February 2023, the epidemiological status in the WHO African Region remained constant. Compared to the pandemic’s previous three years, there were fewer cases, fatalities, and hospitalizations [3]. A COVID-19 vaccine is designed to offer acquired immunity against severe acute respiratory syndrome coronavirus 2 (SARS-CoV-2), the virus that causes coronavirus illness 2019 (COVID-19), in an effort to decrease the number of cases and fatalities that have been reported. Globally, 64% of people have received their full primary series of vaccinations, but only 21% of those in low-income countries and 28% of those in Africa do [4].

To achieve high vaccination uptake and herd immunity, public acceptability and confidence in COVID-19 vaccines must be ensured [3, 5]. However, the accelerated development and issue process of COVID-19 vaccines may increase public concerns regarding their safety and effectiveness [6]. The anti-vaccine movement, the COVID-19 vaccine’s politicization, and the disease’s novelty could all have a detrimental impact on vaccine adoption [7].

There is significant global variation in the public’s reception of COVID-19 vaccinations, according to prior studies [8–23]. Vaccine is a complex and context specific issue that varies across time, place, and vaccines. With the evolution of the pandemic and widespread dissemination of COVID-19 related misinformation [24], public acceptance may change over time. Few research has carefully analyzed and synthesized the available evidence, despite the fact that a growing body of work has examined public acceptance of COVID-19 immunization. The acceptance level of the COVId-19 vaccine has not yet been the subject of a systematic review. However, the results of this review’s projections are mixed, making it challenging to recommend actions to support global COVId-19 immunization campaigns. Therefore, umbrella review is needed to pool these scattered results in to a summary estimate of the acceptance level of COVID-19 vaccine in world.

## Objectives

- To assess the pooled COVID-19 vaccine acceptance rate in global context
- To assess the determinants of COVId-19 vaccine acceptance rate in global context

## Methods

This umbrella review was done following the methodology of umbrella review of existing Systematic Review and Meta-analysis (SRM) studies [25]. From checking PROSPERO, this umbrella review wasn’t registered. The protocol for this umbrella review has been submitted for PROSPERO for registration and can be found from the corresponding author on a reasonable request. It was undertaken through systematic synthesis of the eligible Systematic Review and Meta-analysis (SRM) reports on COVID-19 vaccine acceptance rate and its predictors in the world.

### Searching strategy and information sources

We identified studies providing data on the prevalence of and potential risk factors of COVID-19 vaccine acceptance rate from PubMed, Embase, Scopus, Web of Sciences, Cochrane Database of Systematic Reviews, Scopus and Google Scholar were searched Systematic Review and Meta-analysis (SRM) which reported COVID-19 Vaccine acceptance and/or its determinants in global context using PICO frameworks. The search included MeSH terms and keywords, combinations, and snowball searching in references list of articles found through the data base search to retrieve additional articles. For each condition, five concepts and key search terms were identified and used to develop search strategies. Concept 1: (COVID-19): ‘COVID-19’, ‘coronavirus’, ‘corona virus’, ‘coronavirus 19’, ‘SARS CoV 2’, ‘global pandemic’, ‘novel coronavirus’, and coronavirus infection. Concept 2 (Vaccine): ‘vaccine’, ‘inoculate’, ‘immunize’, ‘injection’, and ‘shot’. Concept 3 (Hesitancy): ‘hesitancy’, ‘refuse’, ‘indecision’, ‘acceptance’, ‘uptake’, ‘reluctant’, and ‘skeptic’. Concept 4 (determinants): ‘risk factor’, ‘predictor’, cause’ and determinant’. Concept 5 (SRM): meta-analysis’, ‘systematic review’, and ‘review’. The literature search was done by two reviewers independently, with discrepancy resolved by consensus. Articles with incomplete reported data were handled through contacting corresponding authors. We used the search terms independently and/or in combination using “OR” or “AND” ((‘*COVID-19’OR ‘coronavirus’ OR ‘corona virus’ OR ‘coronavirus 19′ OR ‘SARS CoV 2’ OR ‘global pandemic’ OR ‘novel coronavirus’ OR coronavirus infection) AND (‘vaccine’ OR ‘inoculate’ OR ‘immunize’ OR ‘injection’ OR ‘shot’) AND (‘hesitancy’ OR ‘refuse’ OR ‘indecision’ OR ‘acceptance’ OR ‘uptake’ OR ‘reluctant’ OR and ‘skeptic’) AND (‘risk factor’ OR ‘predictor’ OR cause’ OR determinant’) AND (meta-analysis’ OR ‘systematic review’ OR ‘review’*).

A sample of the literature search strategy, PubMed search strategy, developed using a combination of MeSH terms and free texts is presented as a supplementary file (Supplementary Table 1). In addition to the systematic database searching, article searching was done using the reference list of the included studies and the ‘cited by’ and ‘related articles’ function of PubMed.

### Study selection / eligibility criteria

Retrieved Systematic Review and Meta-analysis (SRM) were exported to reference manager software, Endnote version 8 to remove duplicate studies. Two investigators (BB and BD) independently screened the selected studies using their titles and abstracts before retrieval of full-text papers. We used pre-specified inclusion criteria to further screen the full-text articles. Those Systematic Review and Meta-analysis (SRM) had reported the prevalence and/or at least one associated factors of COVID-19 vaccine acceptance rate and published in English language in global context. For a study to be considered as systematic review or meta-analysis, it should have to meet the following predefined criteria: (a) presented a defined literature search strategy, (b) appraised included studies using a relevant tool, and (c) followed a standard approach in pooling studies and providing summary estimates. Studies were excluded due to any of the following reasons: (a) no report on the measures of interest for this study, (b) language other than English, and (c) narrative reviews, editorials, correspondence, abstracts, and methodological studies. The screening and selection of studies was conducted in two stages. First, title and abstract screening was done and then, full-text reviewing was done. Disagreements were discussed during a consensus meeting with other reviewers for the final selection of studies to be included in the umbrella review.

## Quality assessment

Methodological quality of all included reviews were assessed by two independent reviewers using the Assessment of Multiple Systematic Reviews (AMSTAR) tool [26, 27]. It consists of 11 questions that measure the quality of the approaches used for pooling the empirical studies included in the review and summarizing their estimates. The tool has been validated and frequently used for appraisal of the quality of Systematic Review and Meta-analysis (SRM) works. The quality scoring was done out of 11, with scores 8–11, 4–7, and < 3 indicating high, medium, and low qualities, respectively. The decision as to whether or not to include a review can be made based on meeting a pre-determined proportion of all criteria, or on certain criteria being met. Decisions about a scoring system or any cut-off for exclusion was made in advance and agreed upon by all reviewers before critical appraisal commences. We have checked the quality of the included primary/ original research studies in each of the research syntheses that have been included in the umbrella review.

### Data extraction

Data from the included Systematic Review and Meta-analysis (SRM) studies were extracted using a standardized data abstraction form, developed in excel spreadsheet. For each Systematic Review and Meta-analysis (SRM) study, the following data were extracted: (a) identification data (first author’s last name and publication year), (b) Review aim and type (c) COVID-19 acceptance (%) (d) risk factors for COVID-19 acceptance (%) (e) odds ratio or relative risk with 95% confidence intervals for COVID-19 acceptance (%), (f) number of primary studies included within each Systematic Review and Meta-analysis (SRM) study and their respective design type, (g) total number of sample size included, (h) publication bias assessment methods and scores, (i) quality assessment methods and scores, (j) data synthesis methods (random or fixed-effects model), and (k) the authors’ main conclusion of the Systematic Review and Meta-analysis (SRM) study (Table 1).

**Table 1:** Distribution of included reviews on the global COVID-19 vaccine acceptance level and its determinants, 2023

Sr No	Authors	Year	Country	Study design	Number of articles included	Sample size	COVID-19 acceptance (%)
**1.**	Sahile, A.T., et al., [9]	2022	Ethiopia	SR and MA	18	10,873	57.8
**2.**	Wake, A.D., [10]	2021	Africa	SR and MA	22	33,912	48.93
**3.**	Alemayehu, A., et al., [11]	2022	East Africa	SR and MA	25	33,044	60.2
**4.**	Wake, A.D. [28],	2021	Global	SR	45	577,835	
**5.**	Mose, A., et al., [12]	2022	Ethiopia	SR and MA	12	5029	51.64
**6.**	Desye, B [29],.	2022	Global	SR	33		
**7.**	Mengistu, D.A., [13]	2022	Global	SR and MA	68	6773	64.9
**8.**	Belay, G.M., et al. [14],	2022	Ethiopia	SR and MA	14	6773	51.2
**9.**	Yehualashet, D.E., et al., [30]	2022	Ethiopia	SR	20	10,277	–
**10.**	Yasmin, F., et al., [31]	2021	United States	SR	65	7,035,448	–
**11.**	Gudayu, T.W [32].	2023	Sub-Saharan African	SR and MA	35	37,345	–
**12.**	Akem Dimala, C., et al., [15]	2021	Global	SR and MA	35	70,997	71
**13.**	Norhayati, M.N., [16]	2022	Global	SR and MA	172	832,709	61
**14.**	Jarrett, C., et al., [33]	2014	Global	SR	14		–
**15.**	Wang, Q., et al., [17]	2021	Global	SR and MA	38	33,844	73.31
**16.**	Kukreti, S., et al., [18]	2022	Global	SR and MA	19	5981	60.1
**17.**	Moltot, T., et al., [19]	2023	Ethiopia	SR and MA	11	56,913	54.59
**18.**	Nindrea, R.D., et al., [20]	2021	Global	SR and MA	10	9287	62.66
**19.**	Olu-Abiodun, O [21].	2022	Global	SR and MA	10	9287	57.89
**20.**	Mahmud, S., et al., [22]	2021	Global	SR and MA	77	1,581,562	61.74
**21.**	Nehal, K.R., et al. [23],	2021	Global	SR and MA	63	75,417	66.01
**22.**	Shakeel, C.S., et al., [34]	2022	Global	SR	81		–

### Statistical analysis

After the data will be extracted using Microsoft Excel format, we imported the data to STATA version 14.0 statistical software for further analysis. Both narrative and qualitative approaches will be used to summarize the estimates of the included reviews. When two or more estimates were provided on the same topic, we presented the range of the estimates and also calculated a summary (pooled) estimate. Using the binomial distribution formula, Standard error was calculated for each study. We pooled the overall magnitude estimates of pneumonia by a random effect meta-analysis [35]. The pooled prevalence of COVID-19 vaccine acceptance rate with 95% CI was presented using forest plots and Odds ratio (OR) with 95% CI was also presented in forest plot to show the associated factors of COVID-19 vaccine acceptance rate. We examined the heterogeneity between the studies using Cochrane’s Q statistics (Chi-square), invers variance (I^2^) and *p*-values [36]. In this study, the I^2^ statistic value of zero indicates true homogeneity, whereas the value 25, 50, and 75% represented low, moderate and high heterogeneity respectively [27, 37]. For the data identified as heterogeneous, we conducted our analysis by DerSimonian-Laird random-effects model analysis The subgroup analysis was conducted by economic classification of global countries. Sensitivity analysis was employed to see the effect of a single study on the overall estimation. Publication bias was checked by funnel plot and more objectively through Egger’s regression test [38].

## Results

**Fig. 1:**
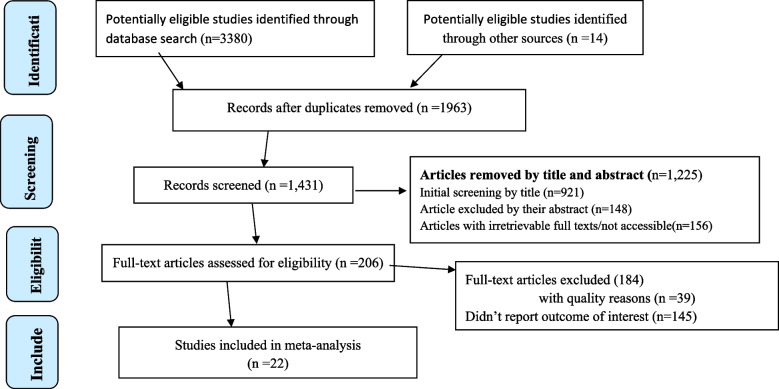
PRISMA –adapted flow diagram showed the results of the search and reasons for exclusion [39]

A total of 3394 reviews were identified; 3380 from different databases and 14 from other sources. After duplication removed, a total of 1431 articles remained (1963 removed by duplication). Finally, 206 studies were screened for full-text review, and 22 Systematic Review and Meta-analysis (SRM) with 10,433,306 were included for the final analysis (Fig. 1, and Supplementary Table 2).

### Characteristics of included studies

Most of the included reviews (16/22) were both systematic review and meta-analysis, while the remaining 6 included studies were only systematic review. Almost all studies (21/22) were published after 2022. All included Systematic Review and Meta-analysis (SRM) included more than 10 studies; The minimum and maximum sample size in these included Systematic Review and Meta-analysis (SRM) were 5029 and 7,035,448 respectively (Table 1).

### COVID-19 vaccine acceptance rate

**Fig. 2:**
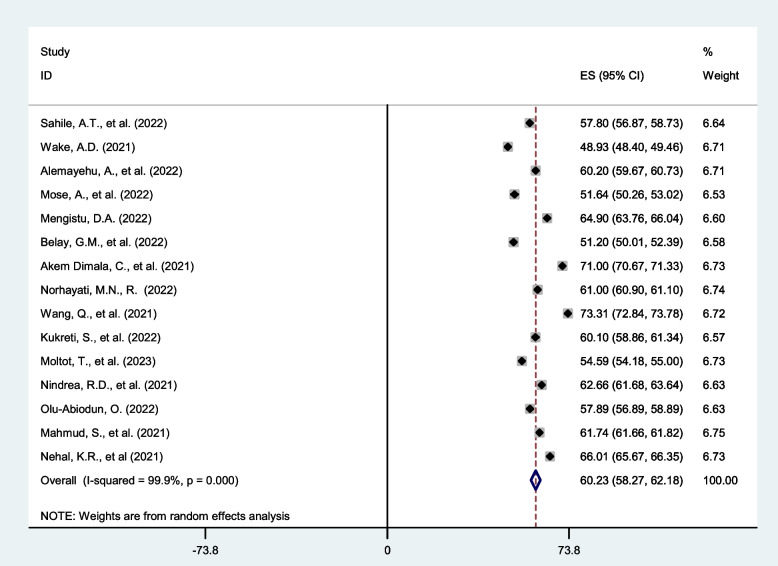
Forest plot shows pooled global acceptance rate of COVID-19 vaccine, 2023

Most of the included studies (*n* = 15) [9–23] have reported COVID-19 vaccine acceptance rate. The magnitude of COVID-19 vaccine acceptance was ranged from 48.93(95% CI: 48.40, 49.46) [28] to 73.31 (95% CI: 72.84, 73.87) [17]. The random-effects model analysis from those studies revealed that, the pooled COVID-19 vaccination acceptance rate globally is found to be 60.23 (95% CI: 58.27, 62.18) (I^2^ = 99·9%; *p* < 0·001) (Fig. 2).

### Assessment of heterogeneity

We used *I*^2^ to assess the heterogeneity of each published SRoMA. *I*^2^ values range from 0 to 100% and are used as a measure of the magnitude of heterogeneity among individual studies and can be used to present the percentage of the total variance caused by heterogeneity [36]. *I*^2^ < 50% can be considered nonsignificant heterogeneity. *τ*^2^ was also calculated, which can explain the between-study variance associated with risk estimates because *I*^2^ is affected by study size [40]. In addition, we calculated a 95% PI for the SHR, which further explains the heterogeneity among studies. This interval with 95% certainty provides a prediction range for the potential true effect size of future studies [40]. The *I*^2^, *τ*^2^, and 95% PI for each published SRoMA were calculated [41]. In this study, the random-effects model analysis from those studies revealed that, the pooled COVID-19 vaccination acceptance rate globally is found to be 60.23 (95% CI: 58.27, 62.18) (I^2^ = 99·9%; *p* < 0·001).

**Fig. 3:**
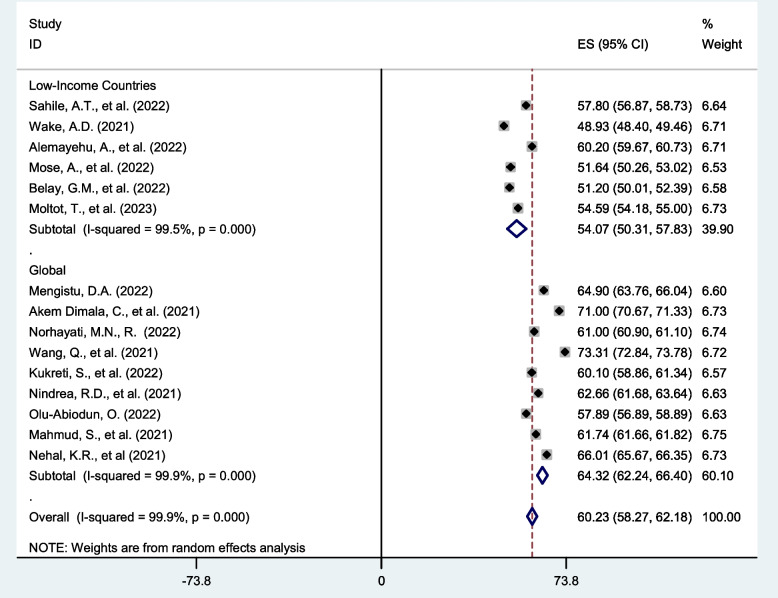
Forest plot shows subgroup analysis of pooled global acceptance rate of COVID-19 vaccine by economic classification of global countries, 2023

Subgroup analysis: Subgroup analysis was done through stratified by economic classification of global countries, and number of included studies. Based on this, the magnitude of COVID-19 vaccine acceptance was found to be 54.07 (50.31, 57.83) in Low income countries, while this magnitude is 64.32 (62.24,66.40) among studies across the globe (Fig. 3).

Publication bias: A funnel plot showed a symmetrical distribution. The Egger’s regression test-value was 0·863, which indicated that, the absence of publication bias. Due to the absence of publication bias, we did not employ a trim and fill analysis (Supplementary Fig. 1). Sensitivity analysis: A leave-one-out sensitivity analysis was employed to identify the impact of the individual study on the pooled acceptance rate of COVID-19 vaccine. The results of this sensitivity analysis showed that the pooled finding were not dependent on a single study. Our pooled estimated acceptance rate of COVID-19 vaccine varied from 59.29 (57.48–61.09) to 61.05 (59.23–62.86) after the deletion of a single study (Supplementary Fig. 2).

### Determinants of COVID-19 vaccine acceptance rate

Out of the total included Systematic Review and Meta-analysis (SRM) five [11, 12, 14, 20, 32] of them revealed the determinants of COVID-19 vaccine acceptance rate. Level of education (AOR ranged from 1.46–3.97), knowledge on COVID-19 (AOR ranged from 1.39–3.36), attitude towards COVID-19 vaccine (AOR ranged from 3.36–5.9), previous history of COVID-19 infection (AOR ranged from 2.7–4.4), male sex (AOR ranged from 1.61–4.46), chronic disease (AOR ranged from 1.47–2.0 was identified as predictors for COVID-19 vaccine acceptance rate globally (Table 2).

**Table 2:** Shows determinants of COVID-19 vaccine acceptance rate in the world, 2023

Factors	Odds ratio(95%CI)		Year	Pooled AOR(95%CI)	I^2^ (*P*-value)
Level of education (Higher)	2.1 (1.37, 2.96)	Alemayehu, A., et al., [11]	2022	1.96 (1.20, 2.73)	56.9% (0.073)
	3.97 (1.94, 8.12)	Mose, A., et al., [12]	2021		
	3.3 (1.7, 6.7)	Belay, G.M., et al., [14]	2022		
	1.46 (1.34, 1.59)	Nindrea, R.D., et al., [20]	2021		
Knowledge on COVID-19	2.1 (1.6, 2.8)	Alemayehu, A., et al., [11]	2022	2.20 (1.36, 3.03)	89.6% (< 0.001)
	3.36 (1.71, 6.61)	Mose, A., et al., [12]	2022		
	2.7 (1.1, 7.1)	Belay, G.M., et al., [14]	2022		
	2.7 (2.3, 3.2)	Gudayu, T.W [32].	2022		
	1.39 (1.29, 1.49)	Nindrea, R.D., et al. [20]	2022		
Attitude towards COVID-19 vaccine	3.8 (2.3, 6.2)	Alemayehu, A., et al. [11]	2021	4.50 (2.89, 6.12)	48.3% (0.144)
	3.36 (1.71, 6.61)	Mose, A., et al., [12]	2023		
	5.9 (4.4, 7.8)	Gudayu, T.W [32].	2021		
Previous history of COVID-19 infection	2.7 (1.6, 4.7)	Alemayehu, A., et al., [11]	2022	3.41 (1.77, 5.06)	40.5% (0.195)
	4.4 (2.8, 6.9)	Gudayu, T.W [32].			
Male sex	1.8 (1.2,2.7)	Alemayehu, A., et al., [11]	2021	1.62 (1.47, 1.77)	0% (0.688)
	4.46 (1.19,16.77)	Mose, A., et al., [12]	2022		
	1.61 (1.47,1.78)	Nindrea, R.D., et al., [20]	2023		
Chronic disease	2 (1.3, 3.1)	Belay, G.M., et al., [14]	2021	1.54 (1.18, 1.90)	22.3% (0.257)
	1.47 (1.31, 1.65)	Nindrea, R.D., et al., [20]	2022		

### Level of education

**Fig. 4:**
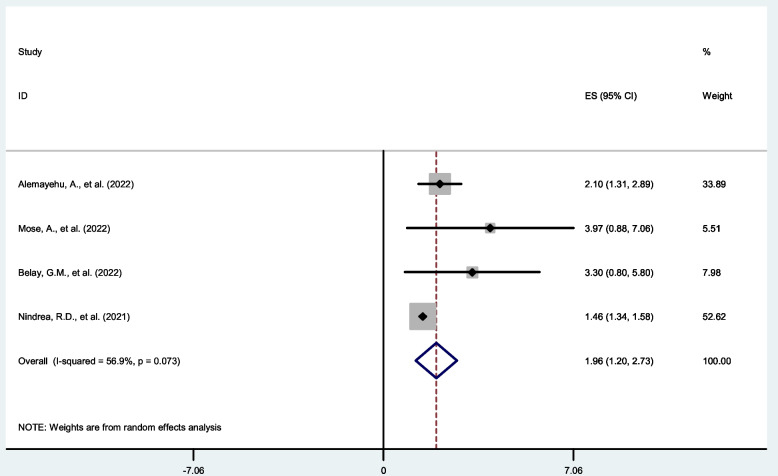
Forest plot shows the pooled estimate of level education as predictor of global acceptance rate of COVID-19 vaccine, 2023

Four SR and MA reported a significant association between level of education and COVID-19 vaccine acceptance rate. Of these the highest risk factor, AOR = 3.97 (95% CI:1.94, 8.12) and lowest risk factor AOR = 1.46 (95% CI:1.34,1.59) among those with respondents with educational level of secondary and above compared to those with educational level primary and illiterate (Table 2). The pooled estimate of AOR of higher level of education was 1.96 (95%C I: 1.20, 2.73; I^2^ = 56.9%; *P* = 0.073) (Fig. 4).

Publication bias: A funnel plot showed asymmetrical distribution. During the Egger’s regression test, the *p*-value was 0.004, which indicated the presence of publication bias (Supplementary Fig. 3); due to this trim and fill analysis was done, and 2 studies were added and the total number of studies become 6. The pooled estimate of AOR of higher level of education becomes 5.48 (2.58,11.61) (Supplementary Fig. 4). To identify the impact of the individual study on the pooled estimate of educational level as a determinant factor for COVID-19 vaccine acceptance rate. The results of this sensitivity analysis showed that our findings were not dependent on a single study (Supplementary Fig. 5).

### Knowledge on COVID-19

**Fig. 5:**
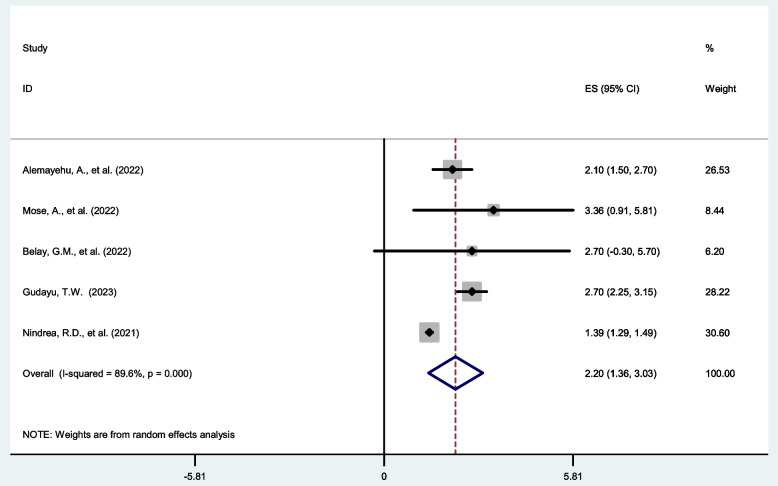
Forest plot shows the pooled estimate of level knowledge on COVID-19 as predictor of global acceptance rate of COVID-19 vaccine, 2023

Five SR and MA reported a significant association between level of knowledge on COVID-19 and COVID-19 vaccine acceptance rate. Of these the highest risk factor, AOR = 3.36 (95% CI:1.71,6.61) and lowest risk factor AOR = 1.39 (95% CI:1.29, 1.49) among those with respondents with good knowledge on COVID-19 compared to those with poor knowledge (Table 2). The pooled estimate of AOR of good knowledge on COVID-19 was 2.20 (95%C I: 1.36, 3.03; I^2^ = 89.6%; *P* = < 0.001) (Fig. 5).

Regarding publication bias, a funnel plot showed a symmetrical distribution. During the Egger’s regression test, the *p*-value was 0.142, which indicated the absence of publication bias (Supplementary Fig. 6); due to this trim and fill analysis was not done.

To identify the impact of the individual study on the pooled estimate of knowledge on COVID-19 as a determinant factor for COVID-19 vaccine acceptance rate. The results of this sensitivity analysis showed that our findings were not dependent on a single study (Supplementary Fig. 7).

### Attitude towards COVID-19 vaccine

**Fig. 6:**
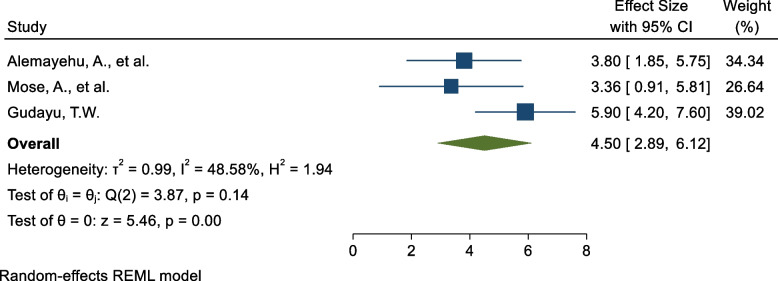
Forest plot shows the pooled estimate of level attitude towards COVID-19 as predictor of global acceptance rate of COVID-19 vaccine, 2023

Three SR and MA reported a significant association between level of attitude towards COVID-19 vaccine and COVID-19 vaccine acceptance rate. Of these the highest risk factor, AOR = 5.9 (95%CI: 4.4,7.8) and lowest risk factor AOR = 3.36 (95%CI:1.71, 6.61) among those with respondents with good attitude towards COVID-19 vaccine compared to those with poor attitude (Table 2). The pooled estimate of AOR of good attitude towards COVID-19 was 4.50 (95%C I: 2.89, 6.12; I^2^ = 48.3%; *P* = 0.144) (Fig. 6).

Regarding publication bias, a funnel plot showed a symmetrical distribution. During the Egger’s regression test, the *p*-value was 0.348, which indicated the absence of publication bias (Supplementary Fig. 8); due to this trim and fill analysis was not done. To identify the impact of the individual study on the pooled estimate of level of attitude towards on COVID-19 as a determinant factor for COVID-19 vaccine acceptance rate. The results of this sensitivity analysis showed that our findings were not dependent on a single study (Supplementary Fig. 9).

### Previous history of COVID-19 infection

**Fig. 7:**
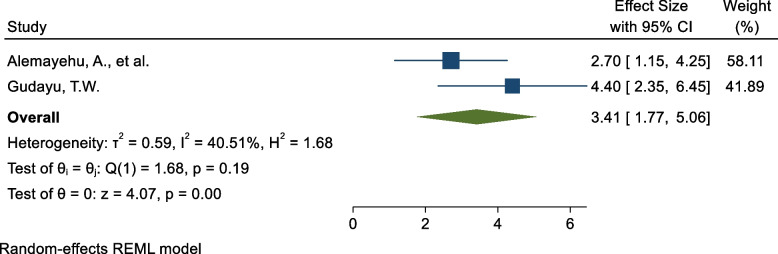
Forest plot shows the pooled estimate of previous history of COVID-19 infection as predictor of global acceptance rate of COVID-19 vaccine, 2023

Two SR and MA reported a significant association between having previous history of COVID-19 infection and COVID-19 vaccine acceptance rate. The pooled estimate of AOR of having previous history of COVID-19 infection was 3.41 (95%C I: 1.77, 5.06; I^2^ = 40.5%; *P* = 0.195) (Fig. 7) as a determinant for COVID-19 vaccine acceptance rate compared to those who had not history of COVID-19 infection. To identify the impact of the individual study on the pooled estimate of previous history of COVID-19 infection as a determinant factor for COVID-19 vaccine acceptance rate. The results of this sensitivity analysis showed that our findings were not dependent on a single study (Supplementary Fig. 10).

### Male sex

**Fig. 8:**
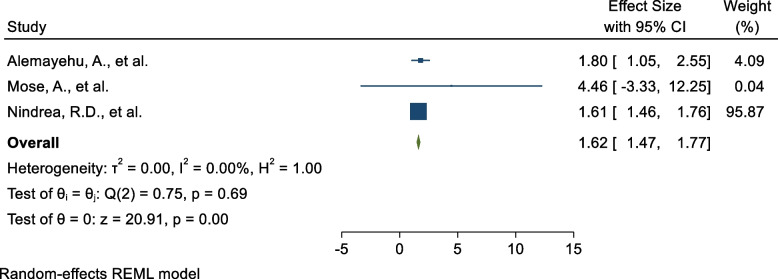
Forest plot shows the pooled estimate of male sex as predictor of global acceptance rate of COVID-19 vaccine, 2023

Three SR and MA reported a significant association between being male and COVID-19 vaccine acceptance rate. The pooled estimate of AOR of being male was 1.62 (95%C I: 1.47, 1.77; I^2^ = 0%; *P* = 0.688) (Fig. 8) as a determinant for COVID-19 vaccine acceptance rate compared to female respondents.

Publication bias: a funnel plot showed a symmetrical distribution. During the Egger’s regression test, the *p*-value was 0.048, which indicated the presence of publication bias (Supplementary Fig. 11); due to this trim and fill analysis was done and 2 studies were added (Supplementary Fig. 12). The pooled estimate becomes 5.003 (4.31,5.80). To identify the impact of the individual study on the pooled estimate of male sex as a determinant factor for COVID-19 vaccine acceptance rate. The results of this sensitivity analysis showed that our findings were not dependent on a single study (Supplementary Fig. 13).

### Chronic disease

**Fig. 9:**
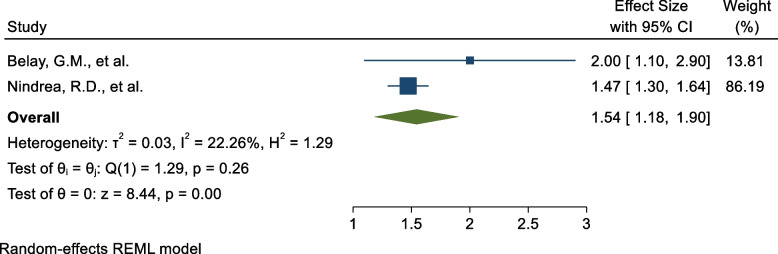
Forest plot shows the pooled estimate of chronic disease as predictor of global acceptance rate of COVID-19 vaccine, 2023

Two SR and MA reported a significant association between having chronic disease comorbidity and COVID-19 vaccine acceptance rate. The pooled estimate of AOR of having chronic disease comorbidity was 1.54 (95%C I: 1.18, 1.90; I^2^ = 22.3%; *P* = 0.257) (Fig. 9) as a determinant for COVID-19 vaccine acceptance rate compared to those without chronic disease.

To identify the impact of the individual study on the pooled estimate of chronic disease as a determinant factor for COVID-19 vaccine acceptance rate. The results of this sensitivity analysis showed that our findings were not dependent on a single study (Supplementary Fig. 14).

### Assessment of excess significance bias

The exploratory test evaluates if factors like publication bias, selective analyses, outcome reporting, or manipulated data contribute to the excess of formally significant findings in published literature. It compares the expected number of studies with statistically significant results to the observed number. The test is best used across multiple meta-analyses with similar characteristics [42–45]. The symmetry of the funnel plot and the Egger’s regression test *P*-value for the COVID-19 acceptance rate and its determinants have both been used by the authors of the current study to assess the small study impacts (publication bias) objectively and subjectively. Thus, 1) for the magnitude, a symmetrical distribution was displayed in a funnel plot. The value of the Egger’s regression test was 0.863, indicating the lack of publication bias. We did not do a trim and fill analysis because there was no publication bias (Supplementary Fig. 1). 2) about the determinants: we performed a trim-and-add study in some of the factors (Supplementary Fig. 3, Supplementary Fig. 4, Supplementary Fig. 11) where a minor study effect was present (with an asymmetric funnel plot and a significant *P*-value in Egger’s regression test) (Supplementary Fig. 6, and Supplementary Fig. 8).

### Prediction intervals

The prediction interval is far more revealing, although it is not as frequently reported [46, 47]. A prediction interval presents heterogeneity on the same scale as original outcomes, whereas a 95% prediction interval estimates true effects for 95% of similar studies. In absence of heterogeneity, the interval coincides with the respective CI. In cases of heterogeneity, the interval covers a wider range than a CI, indicating that conclusions based on CIs may not hold. In the current study, 8 % of them had a 95% prediction interval that excluded the null value.

### Assessment of publication bias and small study effects

Contour-enhanced funnel plots were used to assess publication bias in SRoMAs. Missing studies in areas without significant differences suggest publication bias, while significant differences suggest heterogeneity [38, 48]. Egger’s regression asymmetry test was employed to see if indications of small study effects existed (i.e., small studies provide greater effect size estimates than large studies) [38]. As in prior umbrella reviews [42, 43, 49], a *p* value< 0.10 with a more conservative effect in the largest study (the study with the smallest standard error) than in the random-effects meta-analysis was deemed to be indicative of small study effects. To estimate true effects, the “trim-and-fill” approach was applied when Egger’s test was statistically significant [50]. The contour-enhanced funnel plots, Egger’s test, and the trim-and-fill method were conducted [41, 51]. Small study effects and excess significance bias were found in 30% of studies.

### Credibility assessment

Credibility assessment criteria classify evidence of significant outcomes into four classes: convincing (Class I) highly suggestive (Class II), suggestive (Class III), and weak (Cass IV). In the current umbrella review, about 65% of systematic reviews and meta-analyses showed high to moderate heterogeneity, while 35% had low heterogeneity.

## Discussion

The main aim of this umbrella review was to assess the global COVID-19 vaccine acceptance level and its determinants. This umbrella review investigates the prevalence and determinate of COVID-19 vaccine acceptance rate, and the pooled prevalence COVID-19 vaccination was 60.23 (95% CI: 58.27, 62.18). The study identified several key factors associated with vaccine acceptance rate, such as being male, older ages, attitude toward vaccine, having knowledge about COVID-19, high academic performance or educational level, a history of COVID-19 infection, having chronic disease were the significant factors for COVID-19 vaccine acceptance.

From the current umbrella review, the estimated pooled prevalence for COVID-19 vaccination acceptance rate 60.23 (95% CI: 58.27, 62.18). This pooled prevalence of the highest vaccine acceptance rates was observed than the surveys that were conducted in most African country like Ethiopia (31.4%), Ghana (39.3%), DR Congo (55.9%), in Pakistan, 1% of the population was fully vaccinated and 2.6% was partially vaccinated and Uganda (53.6) in Egypt (13.5%), and South Africa (63.3%), and estimated pooled prevalence for COVID-19 vaccination acceptance rate among Nigerians ranges between 20.0–58.2% were victims of the frailty in their economies and politics [9, 10, 52]. However, there were substantial differences when compared with studies conducted in respondents from China gave the highest proportion of positive responses 88.6% [11], in an Australian study 80 % (80%) [28], in Chile COVID-19 87% [12], Portugal at 90 and 4%, respectively (or, total at 94%), Cuba at 93% (total), Singapore 89% (total), Canada at 84% (total), followed by Italy, Japan, France in between before Vietnam at 79% (total), Brazil at 78% (total), the UK at 76% (total) [29]. Most African countries showed low acceptance, this variation could have resulted from lower COVID-19 mortality rates as compared to the globe, limited access to multiple COVID-19 vaccines, and the slow supply of COVID-19 vaccines into developing nations, as well as a general perception that African nations are less prone to the disease, which gives doubt on the need for additional resources in vaccination programs in these nations.

Various research demonstrates considerable differences in COVID-19 vaccine acceptance between nations due to the influences of various socio-demographic health variables on vaccination acceptance and risk perception. Since the global roll-out, the degrees of economic development and social and political structures in the nations with high vaccination coverage varied widely. A global survey of potential acceptance of a COVID-19 vaccine has shown that differences in acceptance rates including that 69% of participants in the United States were willing to receive COVID-19 vaccination [13], 93.3% of individuals in Indonesia were willing to receive a vaccine [14]; Respondents from China gave the highest proportion of positive responses 88.6% [11], in an Australian study 80 % (80%) [28], in Chile COVID-19 87% [12], Portugal at 90 and 4%, respectively (or, total at 94%), Cuba at 93% (total), Singapore 89% (total), Canada at 84% (total), followed by Italy, Japan, France in between before Vietnam at 79% (total), Brazil at 78% (total), the UK at 76% (total) [29]. However, some developed countries also have low COVID-19 vaccine acceptance rate relatively low anti-SARS-CoV-2 vaccination rate in Russia (47.1%) [30] and in Poland only 27.3% in total have COVID-19 vaccine acceptance [11]. Disbelief, conspiracy theories, and adverse reactions to the vaccine were among the stated reasons why people refused to receive the COVID-19 vaccination. COVID-19 lack of faith in authorities and other stakeholders, fear of the unknown, effectiveness and safety worries, perceived scientific unclear information, low perception of illness risk [11, 30, 31]. Nonetheless, extremely low vaccination rates (at least one dose administered) like Ethiopia (31.4%), Ghana (39.3%), DR Congo (55.9%), in Pakistan, 1% of the population was fully vaccinated and 2.6% was partially vaccinated and Uganda (53.6) in Egypt (13.5%), and South Africa (63.3%), and estimated pooled prevalence for COVID-19 vaccination acceptance rate among Nigerians ranges between 20.0–58.2% were victims of the frailty in their economies and politics [9, 10, 52]. In this context, vaccination against SARS-CoV-2 remains a serious challenge for most countries worldwide, although in contrast to developed countries in countries with low and middle income (LMIC) there is a higher willingness to accept a vaccine, but limited access to them, beside of other factors were the main factor for low vaccination rate [31].

In this umbrella review, higher level of education, good level of knowledge on COVID-19, attitude towards COVID-19 vaccine, previous history of COVID-19 infection, male sex, and having chronic disease were identified factors of COVID-19 vaccine acceptance rate. Aged participants were more likely to accept vaccination than those young age groups [15–20, 33], which could be because they think they are at risk because they have heard that COVID-19 is more frequently complicated in older age groups than in younger and older age groups who do not have access to the internet, protecting them from false information, fake news, and political sagas spreading online that are all for the anti-vaccine movement. These could be the causes of the older individuals’ high vaccination rate. In addition, those with a medium level of education were more likely to accept the COVID-19 vaccine than those with a low level of educational [15, 17–19, 21]. In many instances, educational disparities have a greater impact on people’s willingness to get vaccinated. Different researchers concurred that people with higher levels of education are more likely to consent to COVID-19 vaccination. and a substantial level of vaccination resistance was associated with poor educational levels. Because they have access to more information sources and are more engaged in life events like the COVID-19 immunizations, it’s possible that persons with higher levels of education are more concerned about their health and wellbeing. However, people with some college or less education were less likely to receive a COVID-19 vaccine [22].

Relative to participants without chronic disease, those with chronic disease were more likely have took COVID-19 vaccine [15, 17, 23, 34, 53]. This is a strong correlation with how willing chronic patients are to receive the COVID-19 vaccine. This may be because chronic patients are more likely to experience COVID-19-related morbidity and death as well as be aware of the impact of COVID-19 viruses on public health. Chronic patients may also have one or more chronic diseases, poor functional status, and frequent hospital visits and admissions. They might agree to the COVID-19 vaccine as a result, which increases the probability of COVID-19 infection among chronic patients relative to other population groups. In contrary, some studies found that people with no history of chronic disease were in more favor of vaccination compared to those who did have such a history [27]. Different studies indicated that males were more likely to accept COVID-19 vaccination than females [15, 17, 19, 33]. This might be due to the fact that males are more exposed to public and at increased perceived risk of acquiring the disease. Having good knowledge about the COVID-19 vaccine increases the acceptance rate of the vaccine [18, 23, 34, 53]. Favorable attitude and behavioral scores were also associated positively with vaccine acceptance [15–17, 34], having a history of COVID-19 infection increase the acceptance of COVID-19 vaccine [15, 17, 34, 54]. However another study revealed that, those who have never been infected with COVID-19 were more willing to receive the vaccine [34].

Sociocultural, economic, and political factors also greatly influenced the level of COVID-19 vaccination level and leads to hesitancy. As anti-vaccination movement persisted, vaccination rates continued to fall as disease outbreaks recurred over the globe. For example, the idea that the measles, mumps, and rubella (MMR) vaccine causes autism in children resulted in a decrease in the adoption of the MMR vaccine [55, 56]. Perceived risks and advantages, vaccination-related perceptions, and sociodemographic factors are the most frequent causes of COVID-19 vaccine acceptance, reluctance, and refusal [57]. Social factors affect the vaccine acceptance. People with liberal view expressed the strongest desire to receive the SARS-CoV-2 vaccine, followed by moderates and conservatives [55, 58–60]. Moderates and conservatives were the next groups to declare the strongest desire to receive the SARS-CoV-2 vaccine after liberals [59]. Freeman, Taylor, and Jung investigated social norms and prosocial concerns. They also looked at the motivational underpinnings of vaccine reluctance and the relationships between social factors and vaccine hesitancy [55, 61, 62]. Contextual factors such as beliefs, perceptions, and attitudes towards the vaccine have effect on vaccine hesitancy. More people were likely to acquire the COVID-19 vaccine if they felt more confident and trust in vaccinations [63, 64]. Bertin et al. discovered that views toward vaccines and intentions to vaccinate against COVID-19 were adversely affected by conspiracy theories of all kinds [49]. According to research, there was a positive correlation between the chance of getting the vaccine and both vaccine efficacy and proof of its effectiveness [55, 65–67]. Conversely, due to anxieties about the increased pace of vaccine development, concerns regarding the efficacy of the vaccines were linked to a lower likelihood of receiving them [59, 68, 69]. Health-Related Perceptions also affects vaccine acceptance. There was a positive correlation between the perception of a high risk of COVID-19 infection and the likelihood of obtaining the COVID-19 immunization [55, 64, 69, 70]. People without health insurance had lower vaccination rates, while people with private health insurance had higher vaccination rates [60, 71, 72]. In this situation, understanding the vaccine acceptance rate, the variables influencing COVID-19 vaccination acceptance, and identifying typical obstacles and facilitators for decision-making in this area are crucial elements in developing efficient strategies to increase vaccination rates among the general population. The aim of this umbrella is to ascertain the acceptance rate of the COVID-19 vaccine and its related factors. Several preliminary studies, even various meta and systematic reviews, have been carried out in various parts of the world regarding the acceptance rate of the COVID-19 vaccine and its associated factors, but no comprehensive study has been found to evaluate and summarize their results.

### Strength and limitation of the study

#### Strength of the study

The present umbrella review had many strengths. The study covers a wide range of data sources and includes a substantial number of systematic reviews and metanalyses, providing a comprehensive view of the global COVID-19 vaccine acceptance. The research methodology follows PRISMA guidelines and is robust, ensuring that the included studies meet specific criteria for quality and relevance. The AMSTAR tool is appropriately used for quality assessment. Subgroup analyses by economic classification are conducted, enhancing the study’s depth. The study addresses an important and timely topic, given the ongoing COVID-19 pandemic and the critical role of vaccine acceptance in controlling the disease. The identification of key factors influencing vaccine acceptance, such as education, knowledge, attitude, and previous infection history, provides insights that can inform public health policies and vaccination campaigns.

Our study synthesized evidence from the existing available published systematic review, and meta-analyses. The AMSTAR 2 instrument was employed to assess the methodological quality of the included meta-analyses. We employed credibility assessment criteria by conducting a large number of statistical tests to classify the level of evidence, as in previous umbrella reviews [43, 44, 73–75]. We used not only contour-enhanced funnel plots but also a combination of Egger’s regression asymmetry tests and the trim-and-fill technique to determine whether the asymmetry of the plots was precipitated by publication bias and to assess whether the included meta-analyses reported exaggerated results.

### Limitation of the study

Despite these strengths the study also has some limitations: as the included studies were not from all countries and this may affect the generalizability of the pooled result. This study result is also with high level of heterogeneity despite the authors tried to reduce it through using weighted inverse variance random-effects model to pool the results and subgroup analysis. Missing data in the included meta-analyses prevented us from calculating some metrics, such as small study effects, *I*^2^, 95% PI, and excess significance bias. Therefore, the level of this part of the evidence could not be evaluated. The umbrella review on vaccine acceptance and hesitancy did not include some reviews without pooled figures, potentially limiting the generalizability of the results. Future research, including cohort studies or randomized controlled trials, is needed to address residual confounding factors. The methodological quality of many meta-analyses was graded as low due to the rigorous items of the AMSTAR 2 instrument. The study’s heterogeneity may arise from sociocultural, economic, and political factors influencing vaccine hesitancy in different regions.

## Conclusions and recommendations

This umbrella review revealed the level of COVID-19 vaccine acceptance highly varied and found to be unacceptably low particularly in low-income countries. Higher level of education, good level of knowledge on COVID-19, attitude towards COVID-19 vaccine, previous history of COVID-19 infection, male sex, and having chronic disease were identified factors of COVID-19 vaccine acceptance rate. Therefore, to enhance COVID-19 vaccine acceptance a collaborative effort of global, national, regional and local stakeholders such as policymakers, and vaccine campaign program planners is needed to improve the acceptance rate of COVID-19 vaccine through health education, training about COVID-19 vaccine safety, effectiveness, and benefits had to be given to uneducated segments of the population to improve the willingness of the community.

### Future research outlook

To lessen bias and confounding factors, future research on COVID-19 vaccination acceptability should make use of high-quality methods such as randomized controlled trials or prospective cohort studies. It is important to adhere to the guidelines for performing systematic reviews, which include doing thorough searches and exclusions. It is also advised to address the societal, political, and economic aspects that affect vaccine reluctance in various geographic areas.

### Supplementary Information

**Additional file 1: Supplementary Table 1. **Search strategy used for one of the databases. **Supplementary Table 2.** Methodological quality of the included studies based on the AMSTAR tool. **Supplementary Figure 1.** Shows publication bias for pooled global acceptance rate  of COVID-19 vaccine, 2023. **Supplementary Figure 2.** Shows sensitivity analysis for pooled global acceptance rate  of COVID-19 vaccine, 2023. **Supplementary Figure 3.** Publication bias for estimate of level education as predictor of global acceptance rate of COVID-19 vaccine by economic classification of global countries, 2023. **Supplementary Figure 4.** Trim and fill analysis for estimate of level education as predictor of global acceptance rate of COVID-19 vaccine by economic classification of global countries, 2023. **Supplementary Figure 5.** Sensitivity analysis for estimate of level education as predictor of global acceptance rate of COVID-19 vaccine by economic classification of global countries, 2023. **Supplementary Figure 6.** Publication bias for estimate of level education as predictor of global acceptance rate of COVID-19 vaccine by economic classification of global countries, 2023. **Supplementary Figure 7.** Sensitivity analysis for estimate of level education as predictor of global acceptance rate of COVID-19 vaccine by economic classification of global countries, 2023. **Supplementary Figure 8.** Publication bias for estimate of level attitude towards COVID-19 as predictor of global acceptance rate of COVID-19 vaccine by economic classification of global countries, 2023. **Supplementary Figure 9.** Sensitivity analysis for estimate of level attitude towards COVID-19 as predictor of global acceptance rate of COVID-19 vaccine by economic classification of global countries, 2023. **Supplementary Figure 10.** Sensitivity analysis for estimate of previous history of COVID-19 infection as predictor of global acceptance rate of COVID-19 vaccine by economic classification of global countries, 2023. **Supplementary Figure 11.** Publication bias for estimate of male sex as predictor of global acceptance rate of COVID-19 vaccine, 2023. **Supplementary Figure 12.** Trim and fill analysis for estimate of male sex as predictor of global acceptance rate of COVID-19 vaccine, 2023. **Supplementary Figure 13.** Sensitivity analysis for estimate of male sex as predictor of global acceptance rate of COVID-19 vaccine, 2023. **Supplementary Figure 14.** Sensitivity analysis for estimate of chronic disease as predictor of global acceptance rate of COVID-19 vaccine, 2023.

**Additional file 2. **PRISMA 2020 Checklist.

## Data Availability

The datasets used and/or analyzed during the current study available from the corresponding author (BBA) on reasonable request.
