# Supplementary material for: Global COVID-19 vaccine acceptance level and its determinants: an umbrella review
Source: BMC Public Health. 2024 Jan 2;24:5. doi: 10.1186/s12889-023-17497-4 (PMC10759439; doi:10.1186/s12889-023-17497-4)
Supplement: Supplementary file 1 — Additional file 1: Supplementary Table 1. Search strategy used for one of the databases. Supplementary Table 2. Methodological quality of the included studies based on the AMSTAR tool. Supplementary Figure 1. Shows publication bias for pooled global acceptance rate of COVID-19 vaccine, 2023. Supplementary Figure 2. Shows sensitivity analysis for pooled global acceptance rate of COVID-19 vaccine, 2023. Supplementary Figure 3. Publication bias for estimate of level education as predictor of global acceptance rate of COVID-19 vaccine by economic classification of global countries, 2023. Supplementary Figure 4. Trim and fill analysis for estimate of level education as predictor of global acceptance rate of COVID-19 vaccine by economic classification of global countries, 2023. Supplementary Figure 5. Sensitivity analysis for estimate of level education as predictor of global acceptance rate of COVID-19 vaccine by economic classification of global countries, 2023. Supplementary Figure 6. Publication bias for estimate of level education as predictor of global acceptance rate of COVID-19 vaccine by economic classification of global countries, 2023. Supplementary Figure 7. Sensitivity analysis for estimate of level education as predictor of global acceptance rate of COVID-19 vaccine by economic classification of global countries, 2023. Supplementary Figure 8. Publication bias for estimate of level attitude towards COVID-19 as predictor of global acceptance rate of COVID-19 vaccine by economic classification of global countries, 2023. Supplementary Figure 9. Sensitivity analysis for estimate of level attitude towards COVID-19 as predictor of global acceptance rate of COVID-19 vaccine by economic classification of global countries, 2023. Supplementary Figure 10. Sensitivity analysis for estimate of previous history of COVID-19 infection as predictor of global acceptance rate of COVID-19 vaccine by economic classification of global countries, 2023. Supplementary Figure 11. Publ [file 12889_2023_17497_MOESM1_ESM.docx]

Supplementary Files

# Supplementary Table 1: Search strategy used for one of the databases

| Medline/PubMed | | |  |
| --- | --- | --- | --- |
|  | **Search terms** | |  |
| Group | **Non-MeSH terms** | **MeSH (sub-terms in MeSH)** |  |
| #1 | COVID-19  Coronavirus  Corona virus  Coronavirus 19  SARS CoV 2  Global pandemic  Novel coronavirus  Corona-virus infection | COVID-19 |  |
| #2 | Vaccine  Inoculate  Immunize  Injection  Shot | Vaccine |  |
| #3 | Hesitancy  Refuse  Indecision  Acceptance  Uptake  Reluctant  Skeptic | Acceptance |  |
| #4 | Causes  Determinants  Associated factors  Predictors  Risk factors |  |  |
| #5 | Review  Meta-analysis  Systematic review | Review |  |
|  |  |  |  |
| #1 AND #2 AND #3 AND #4 AND #5 |  |  |  |

(Prevalence OR magnitude OR epidemiology) AND (causes OR determinants OR associated factors OR predictors OR risk factors) AND (children [MeSH Terms] OR under five OR child OR childhood) AND (pneumonia [MeSH Terms] OR respiratory tract infection) AND Eastern Africa

***Supplementary Table 2:*** Methodological quality of the included studies based on the AMSTAR tool.

| **Author** | **Quality assessment questions** | | | | | | | | | | |  |  |  |
| --- | --- | --- | --- | --- | --- | --- | --- | --- | --- | --- | --- | --- | --- | --- |
|  | Q1 | Q2 | Q3 | Q4 | Q5 | Q6 | Q7 | Q8 | Q9 | Q10 | Q11 | Yes Total | Quality status | Overall appraisal |
| Sahile, A.T., et al.,([1](#_ENREF_1)) | Y | Y | N | Y | Y | N | Y | Y | N | Y | Y | 8/11 | High | Included |
| Wake, A.D., ([2](#_ENREF_2)) | Y | Y | Y | Y | N | Y | Y | Y | Y | N | Y | 9/11 | High | Included |
| Alemayehu, A., et al., ([3](#_ENREF_3)) | Y | Y | N | Y | Y | N | Y | Y | N | Y | Y | 8/11 | High | Included |
| Wake, A.D.,([4](#_ENREF_4)) | Y | Y | Y | Y | Y | N | Y | Y | Y | Y | Y | 10/11 | High | Included |
| Mose, A., et al., ([5](#_ENREF_5)) | Y | N | Y | Y | Y | N | Y | Y | Y | Y | Y | 9/11 | High | Included |
| Desye, B.,([6](#_ENREF_6)) | Y | N | Y | Y | Y | N | Y | Y | Y | Y | Y | 9/11 | High | Included |
| Mengistu, D.A., ([7](#_ENREF_7)) | N | Y | Y | Y | Y | N | Y | Y | Y | Y | Y | 10/11 | High | Included |
| Belay, G.M., et al.,([8](#_ENREF_8)) | Y | Y | Y | Y | Y | N | Y | Y | N | Y | Y | 9/11 | High | Included |
| Yehualashet, D.E., et al., ([9](#_ENREF_9)) | N | Y | N | Y | Y | N | Y | Y | N | Y | Y | 7/11 | Medium | Included |
| Yasmin, F., et al., ([10](#_ENREF_10)) | Y | Y | Y | N | Y | Y | Y | Y | Y | Y | Y | 10/11 | High | Included |
| Gudayu, T.W.([11](#_ENREF_11)) | Y | Y | N | Y | Y | N | Y | Y | N | Y | Y | 8/11 | High | Included |
| Akem D C., et al., ([12](#_ENREF_12)) | Y | Y | Y | Y | Y | N | Y | Y | Y | Y | Y | 10/11 | High | Included |
| Norhayati, M.N., ([13](#_ENREF_13)) | Y | N | Y | Y | Y | N | Y | Y | Y | Y | Y | 9/11 | High | Included |
| Jarrett, C., et al., ([14](#_ENREF_14)) | Y | N | Y | Y | Y | Y | Y | Y | Y | Y | Y | 10/11 | High | Included |
| Wang, Q., et al., ([15](#_ENREF_15)) | Y | Y | Y | Y | Y | N | Y | Y | N | Y | Y | 9/11 | High | Included |
| Kukreti, S., et al., ([16](#_ENREF_16)) | Y | Y | Y | Y | Y | N | Y | N | Y | Y | Y | 9/11 | High | Included |
| Moltot, T., et al., ([17](#_ENREF_17)) | Y | Y | Y | N | Y | Y | N | Y | Y | Y | N | 8/11 | High | Included |
| Nindrea, R.D., et al., ([18](#_ENREF_18)) | N | Y | Y | Y | Y | N | Y | Y | Y | N | Y | 8/11 | High | Included |
| Olu-Abiodun, O.([19](#_ENREF_19)) | Y | Y | N | Y | Y | N | Y | N | Y | Y | Y | 8/11 | High | Included |
| Mahmud, S., et al., ([20](#_ENREF_20)) | N | Y | N | Y | Y | N | Y | Y | N | Y | Y | 7/11 | Medium | Included |
| Nehal, K.R., et al.,([21](#_ENREF_21)) | Y | Y | Y | N | Y | Y | Y | Y | Y | Y | Y | 10/11 | High | Included |
| Shakeel, C.S., et al., ([22](#_ENREF_22)) | Y | Y | N | Y | Y | N | Y | Y | N | Y | Y | 8/11 | High | Included |
| Key: Y=yes, N=no, UC=unclear, Q=Question  AMSTAR Assessment of Multiple Systematic Reviews.  Q1: A priori design; Q2: Duplicate study selection and data extraction; Q3: Search comprehensiveness; Q4: Inclusion of grey literature; Q5: Included and excluded studies provided; Q6: Characteristics of the included studies provided; Q7: Scientific quality of the primary studies assessed and documented; Q8: Scientific quality of included studies used appropriately in formulating conclusions; Q9: Appropriateness of methods used to combine studies' findings; Q10: Likelihood of publication bias was assessed; Q11: Conflict of interest – potential sources of support were clearly acknowledged in both the systematic review and the included studies. | | | | | | | | | | | | | | |

**Supplementary Figures**


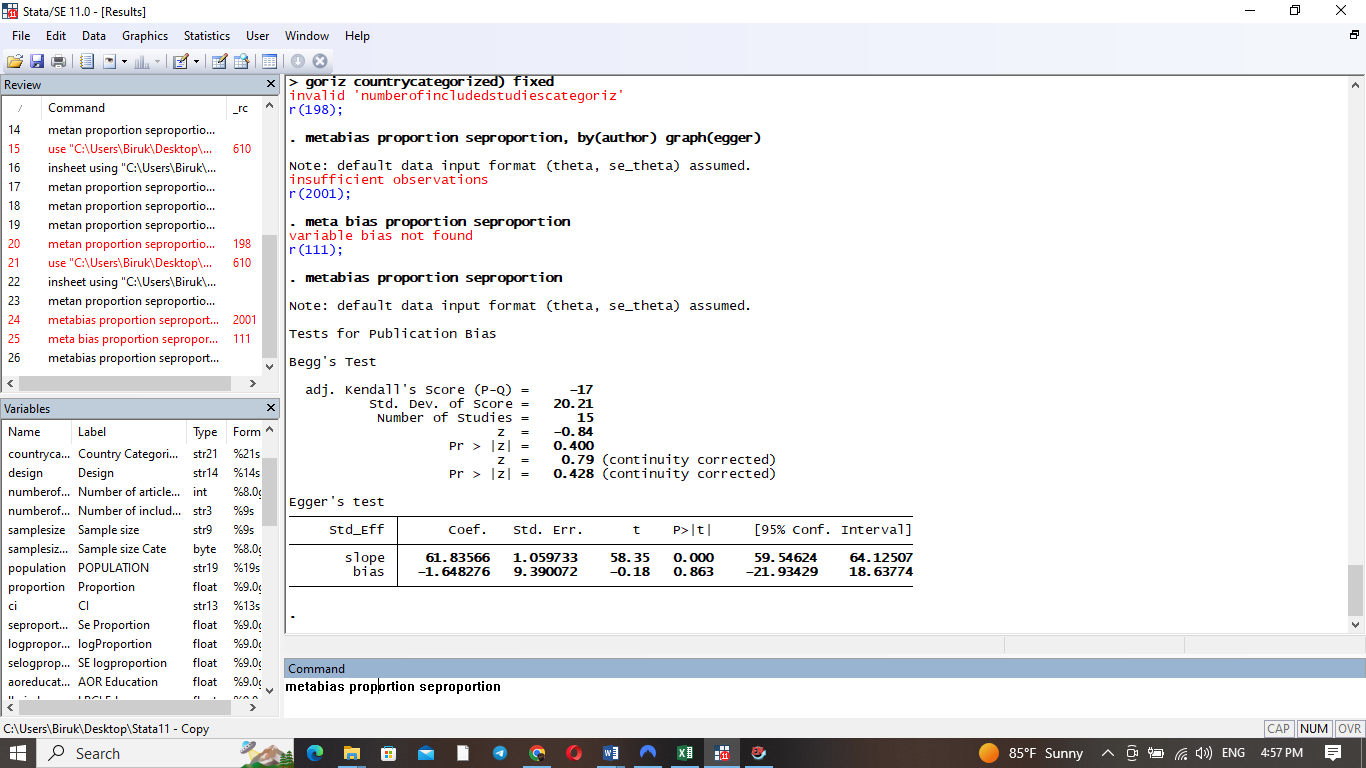


Supplementary Figure 1: Shows publication bias for pooled global acceptance rate of COVID-19 vaccine,2023


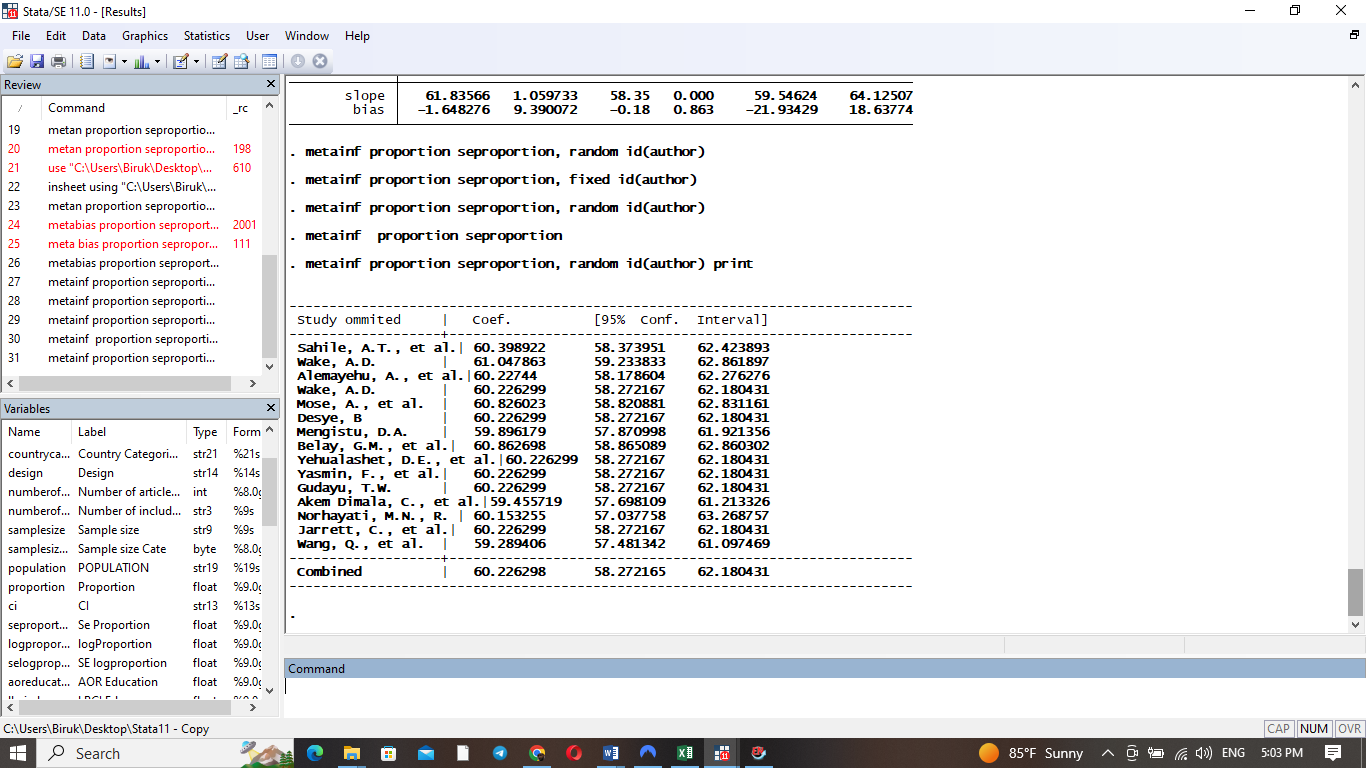


Supplementary Figure 2: Shows sensitivity analysis for pooled global acceptance rate of COVID-19 vaccine, 2023


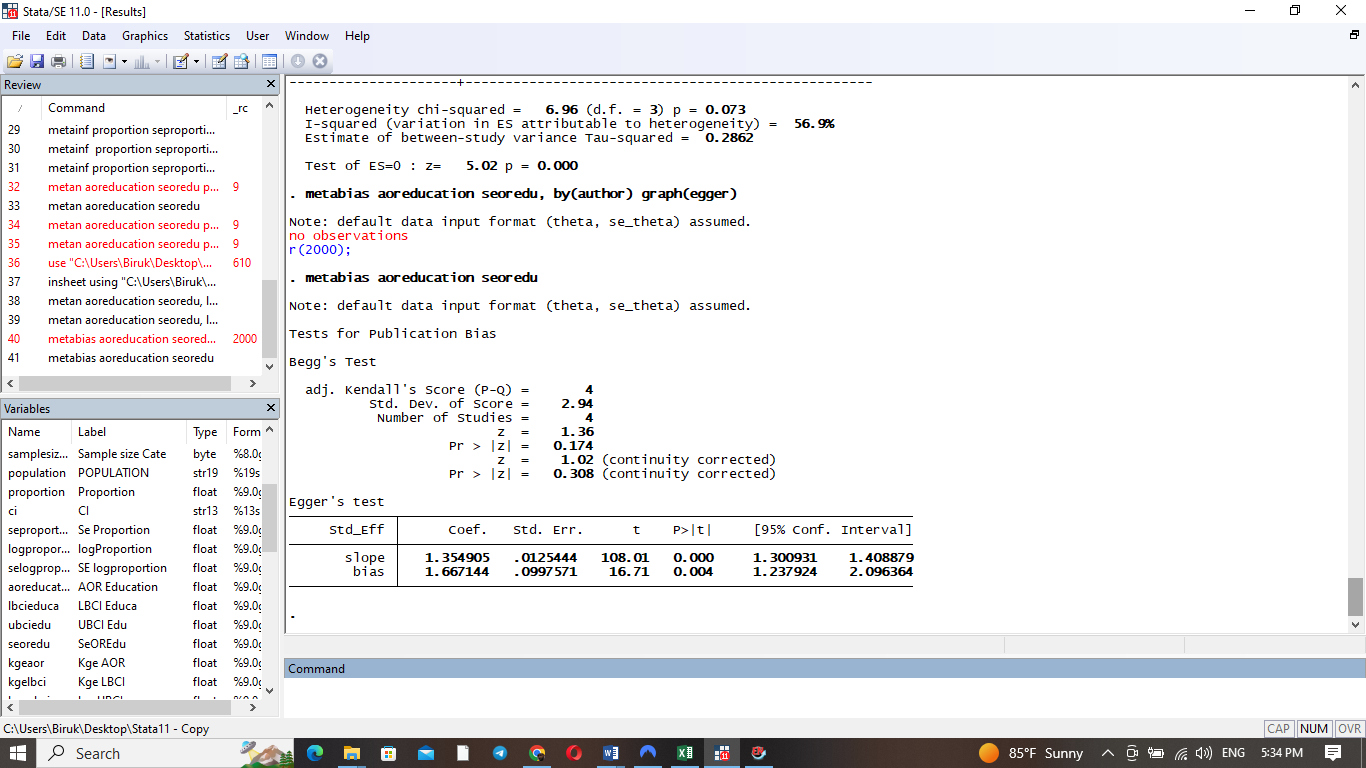


Supplementary Figure 3: Publication bias for estimate of level education as predictor of global acceptance rate of COVID-19 vaccine by economic classification of global countries, 2023


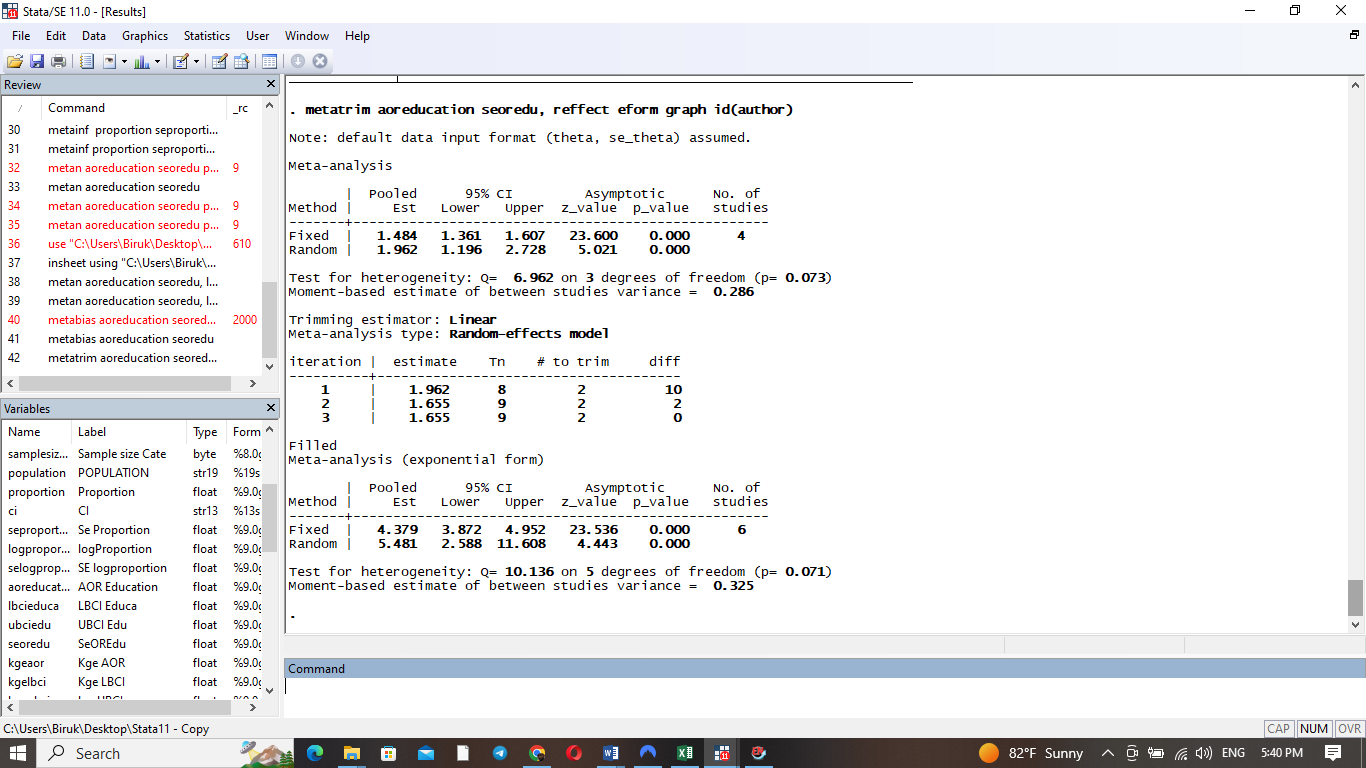


Supplementary Figure 4: Trim and fill analysis for estimate of level education as predictor of global acceptance rate of COVID-19 vaccine by economic classification of global countries, 2023


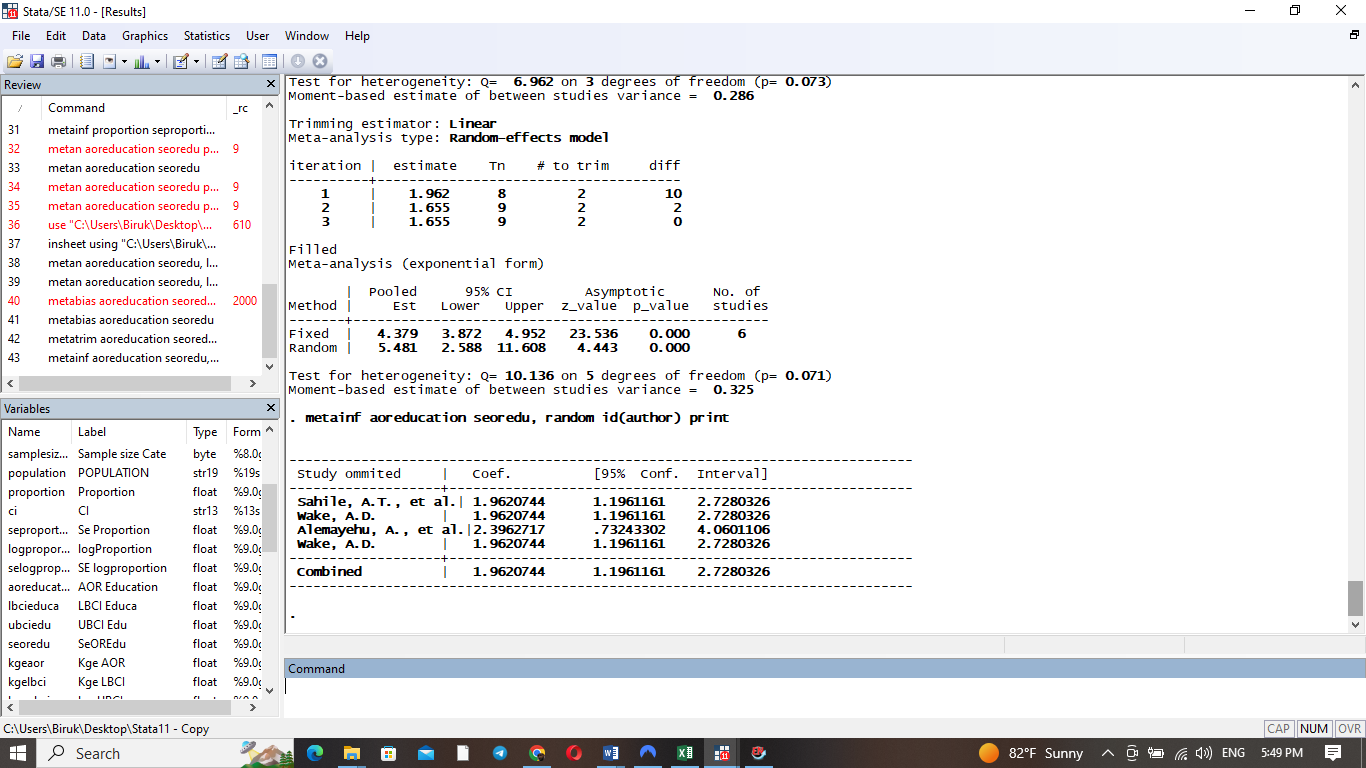


Supplementary Figure 5: Sensitivity analysis for estimate of level education as predictor of global acceptance rate of COVID-19 vaccine by economic classification of global countries, 2023


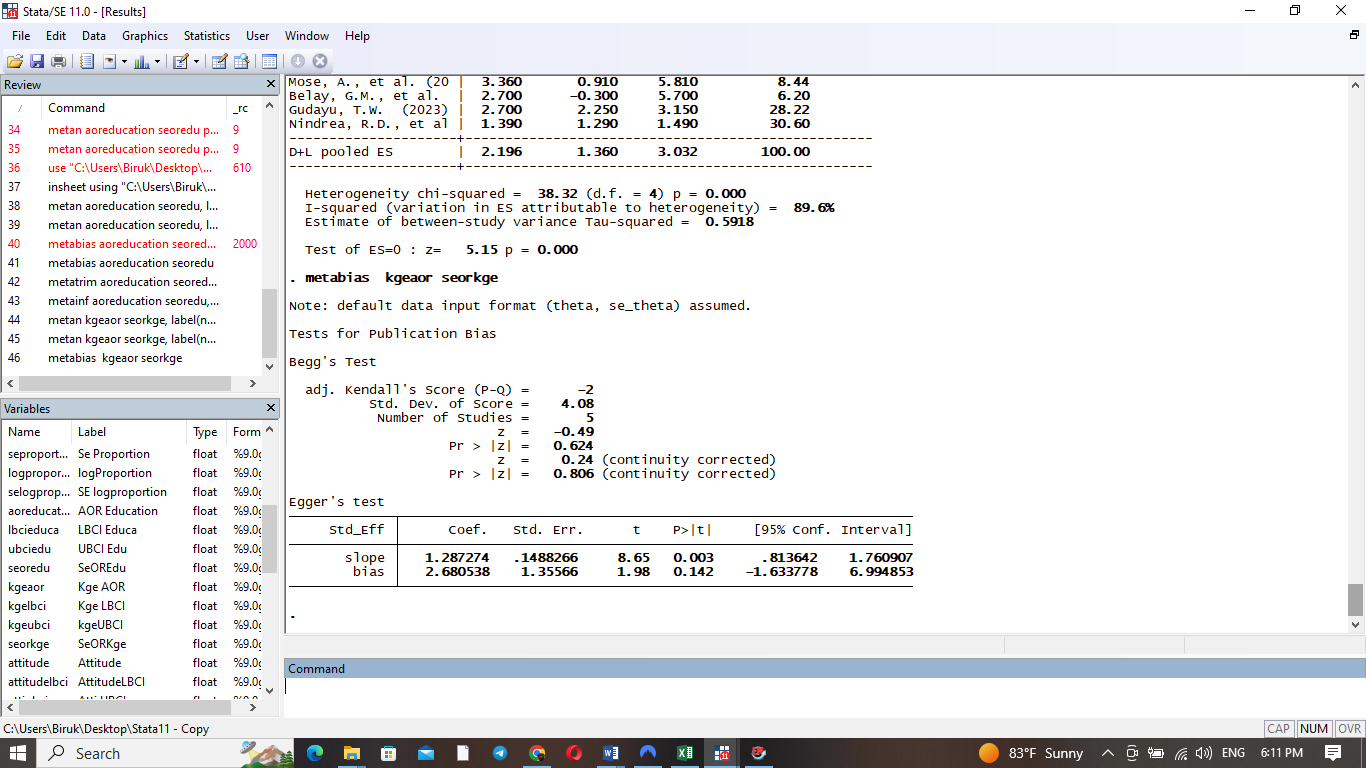


Supplementary Figure 6: Publication bias for estimate of level education as predictor of global acceptance rate of COVID-19 vaccine by economic classification of global countries, 2023


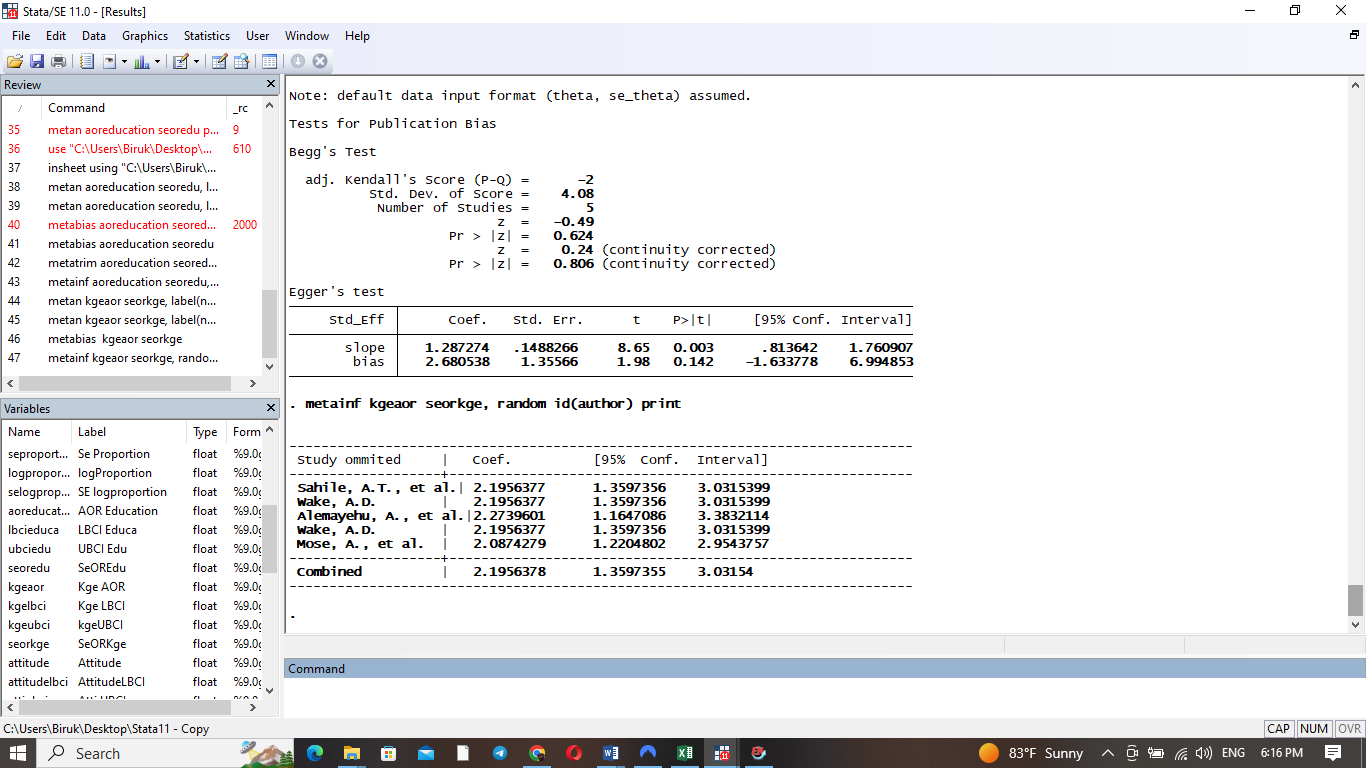


Supplementary Figure 7: Sensitivity analysis for estimate of level education as predictor of global acceptance rate of COVID-19 vaccine by economic classification of global countries, 2023


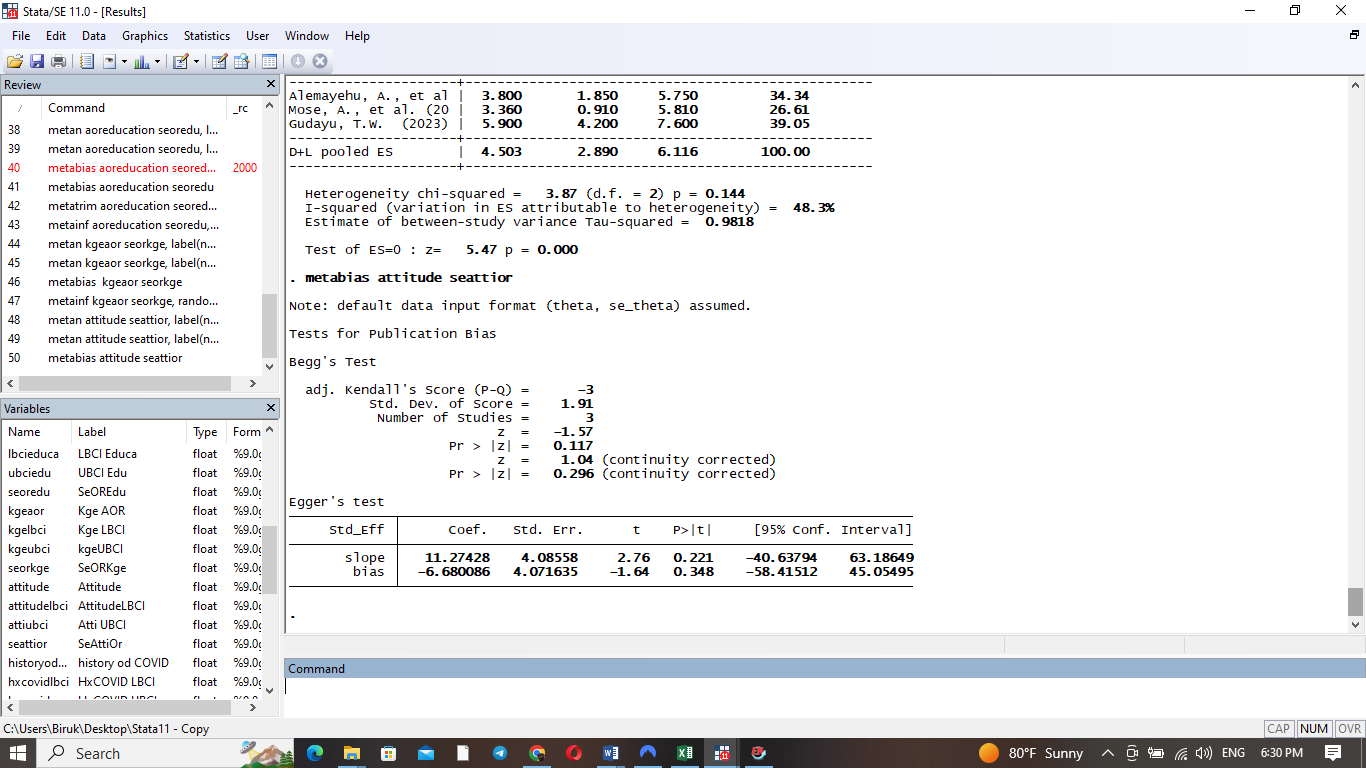


Supplementary Figure 8: Publication bias for estimate of level attitude towards COVID-19 as predictor of global acceptance rate of COVID-19 vaccine by economic classification of global countries, 2023


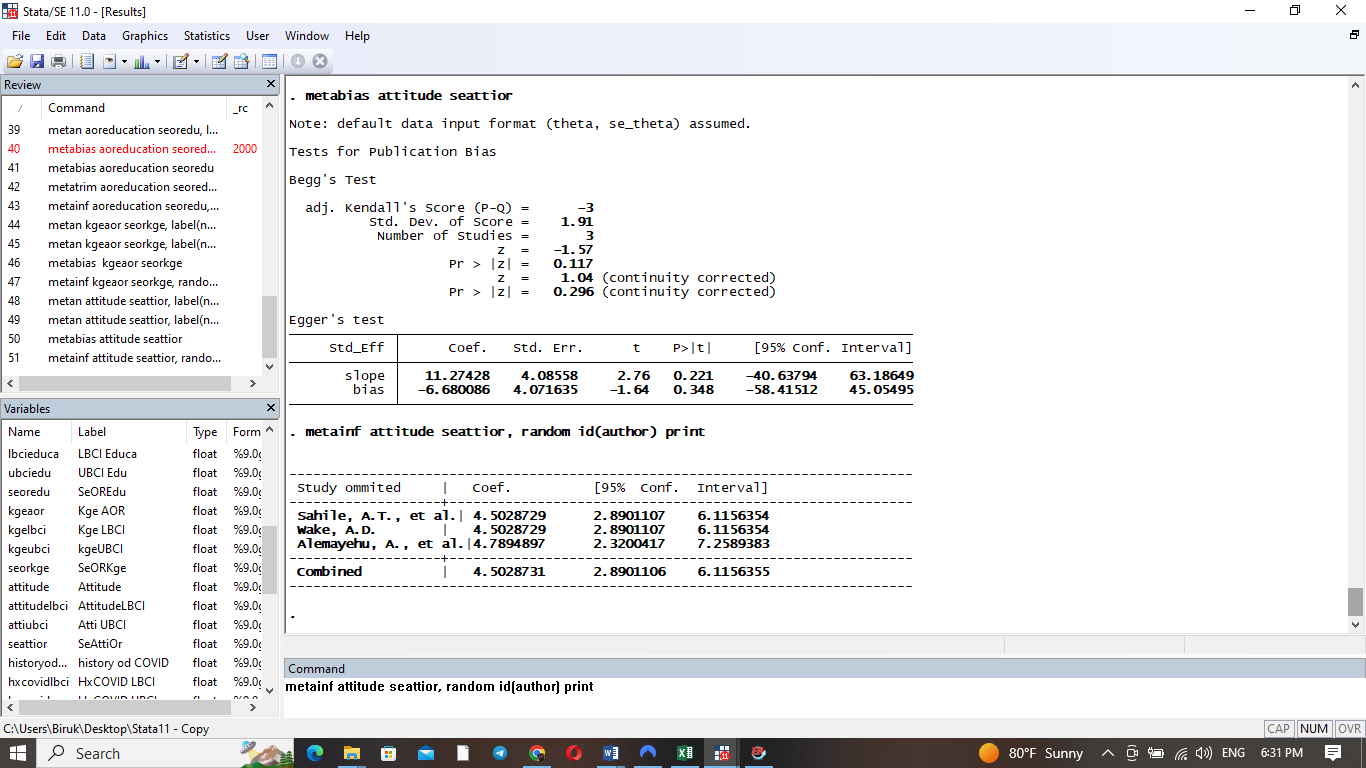


Supplementary Figure 9: Sensitivity analysis for estimate of level attitude towards COVID-19 as predictor of global acceptance rate of COVID-19 vaccine by economic classification of global countries, 2023


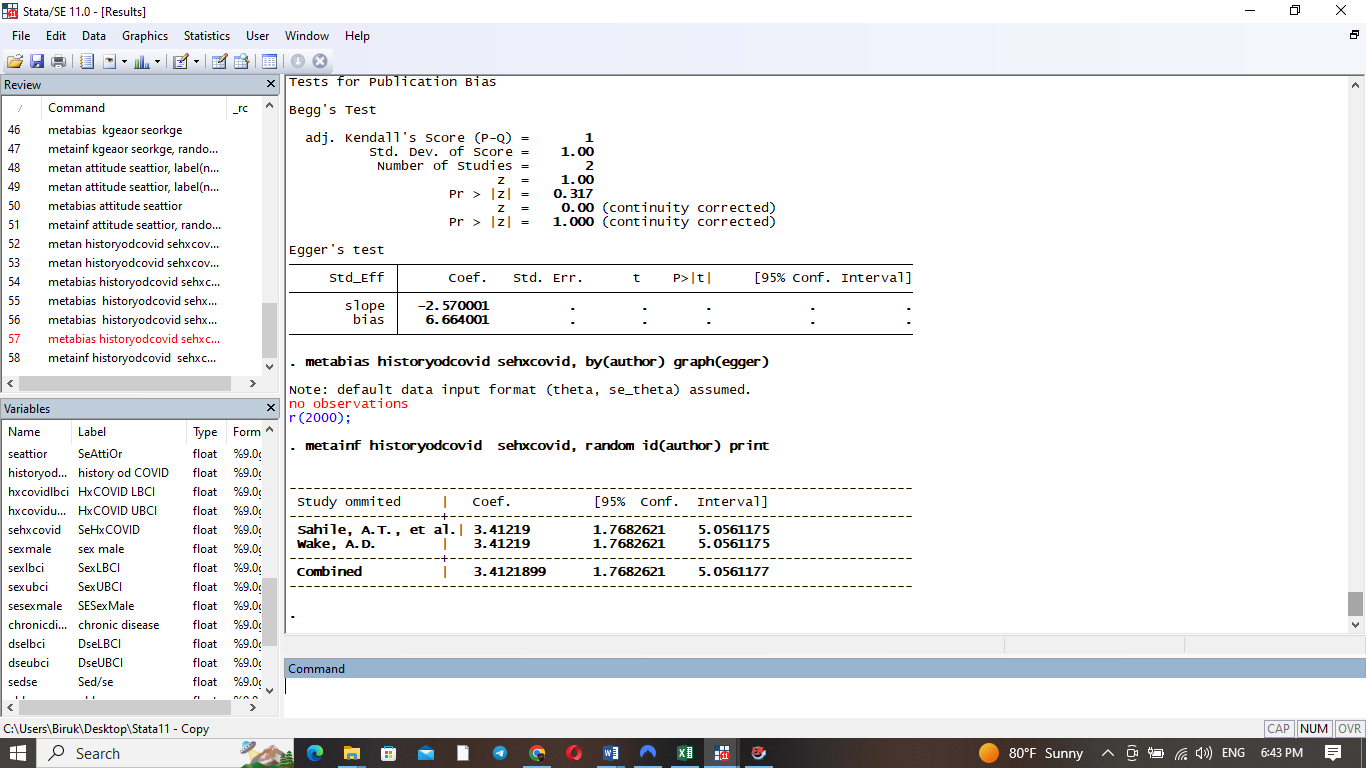


Supplementary Figure 10: Sensitivity analysis for estimate of previous history of COVID-19 infection as predictor of global acceptance rate of COVID-19 vaccine by economic classification of global countries, 2023


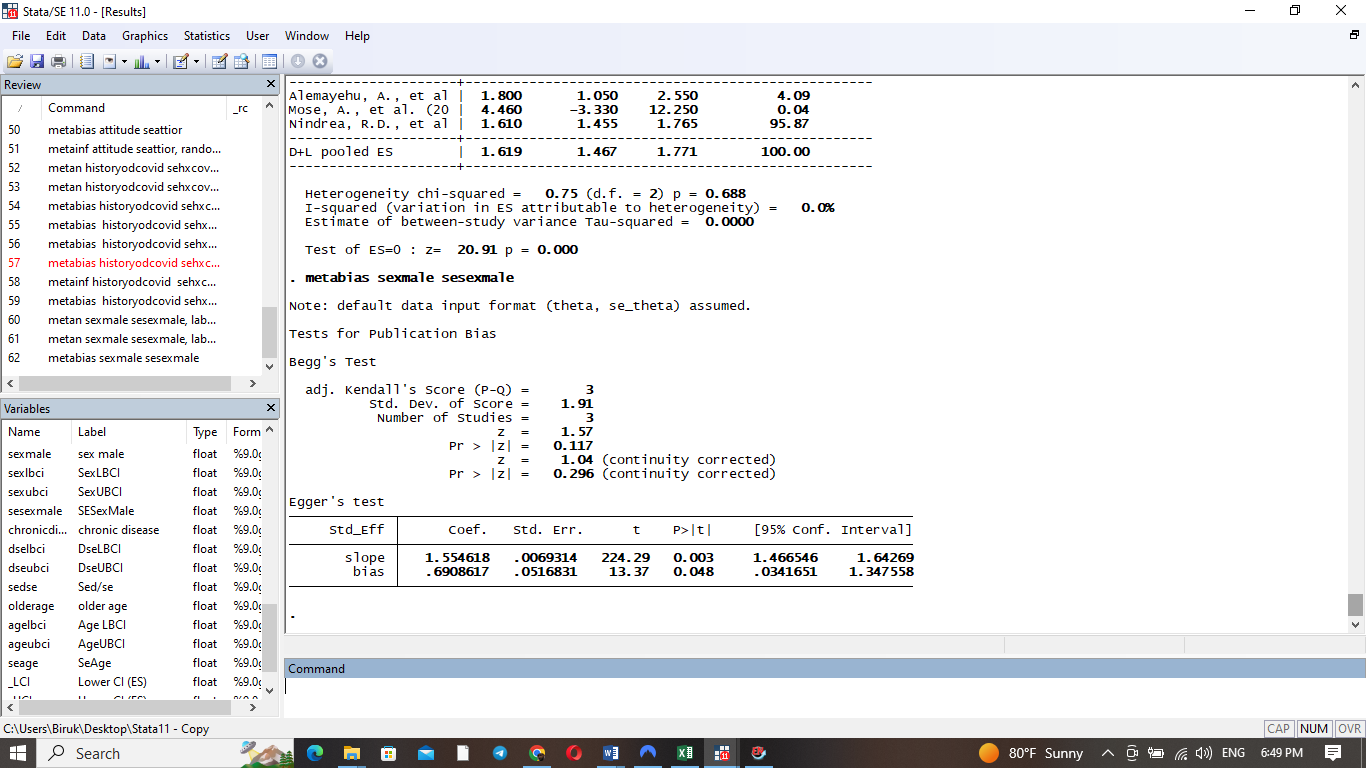


Supplementary Figure 11: Publication bias for estimate of male sex as predictor of global acceptance rate of COVID-19 vaccine, 2023


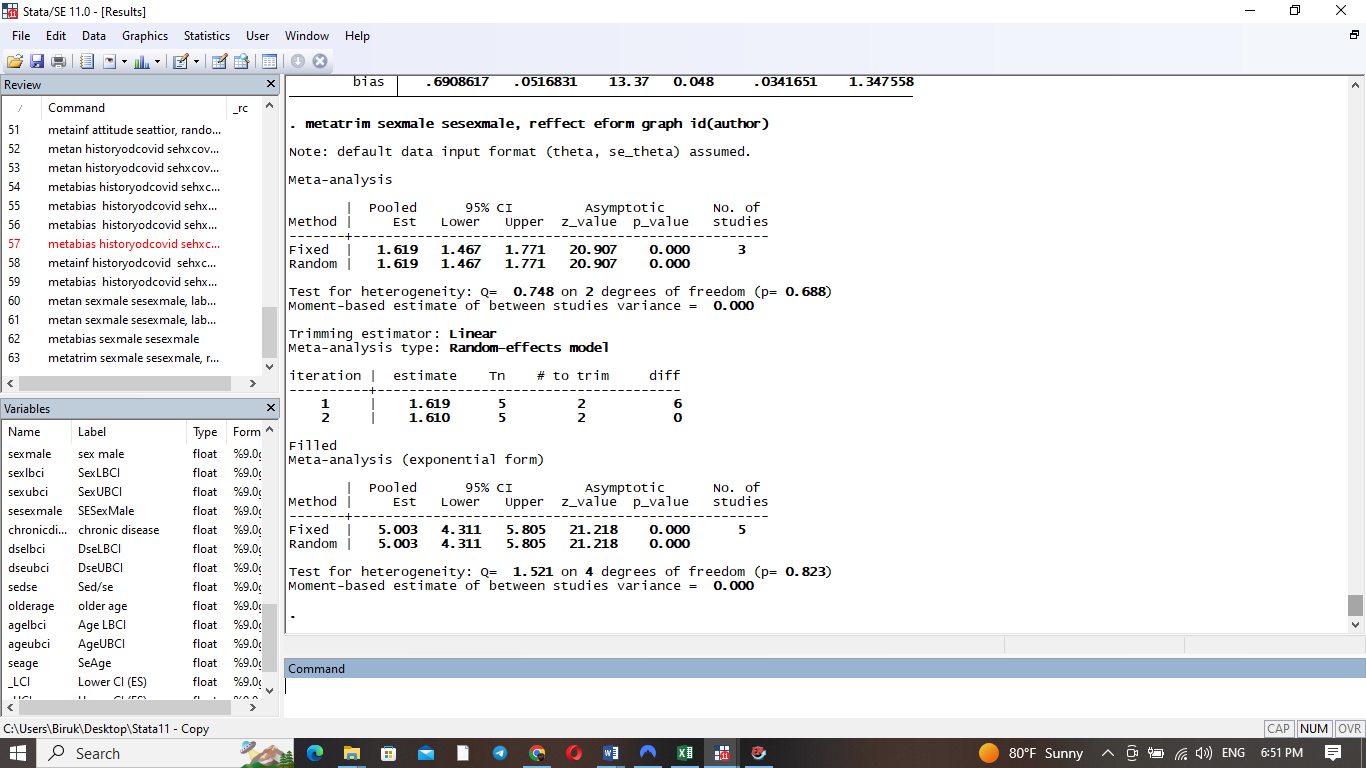


Supplementary Figure 12: Trim and fill analysis for estimate of male sex as predictor of global acceptance rate of COVID-19 vaccine, 2023


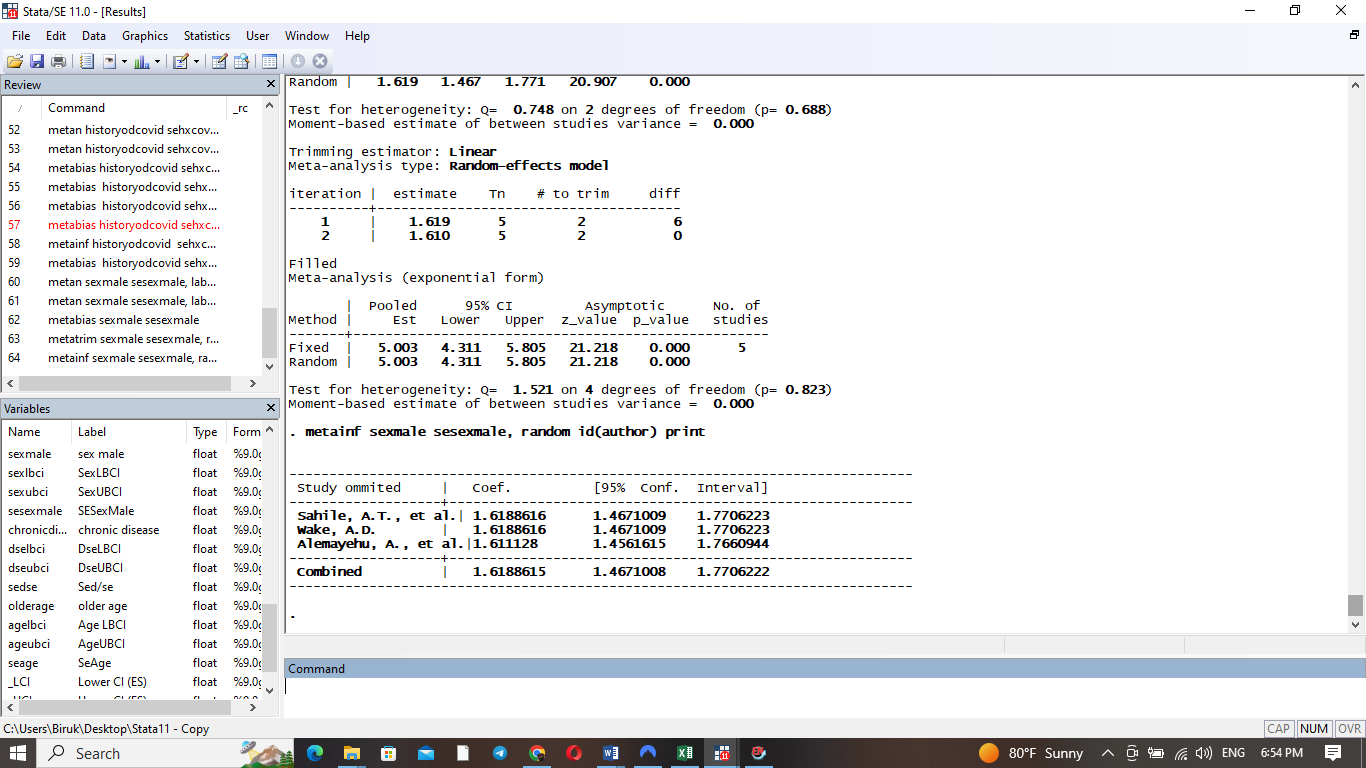


Supplementary Figure 13: Sensitivity analysis for estimate of male sex as predictor of global acceptance rate of COVID-19 vaccine, 2023


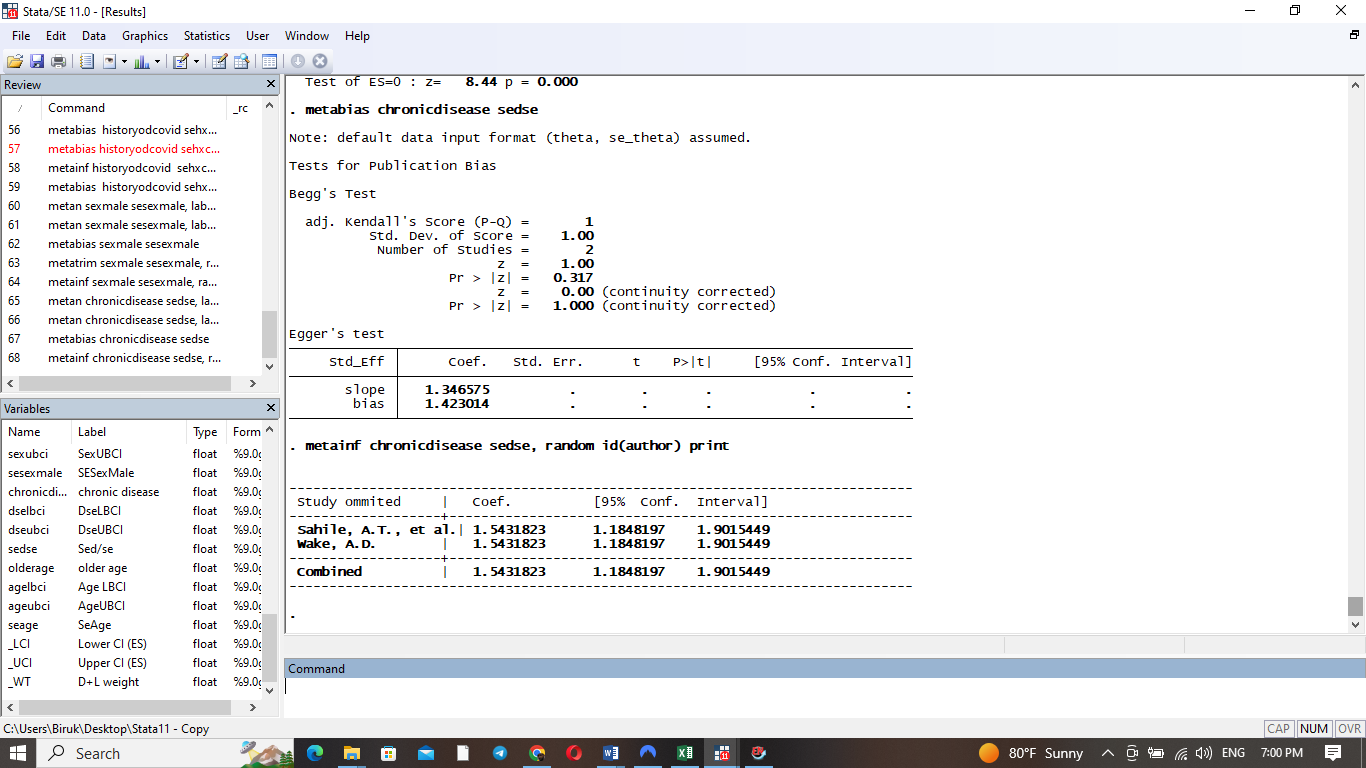


Supplementary Figure 14: Sensitivity analysis for estimate of chronic disease as predictor of global acceptance rate of COVID-19 vaccine, 2023

References

1. Sahile AT, Gizaw GD, Mgutshini T, Gebremariam ZM, Bekele GE. COVID-19 Vaccine Acceptance Level in Ethiopia: A Systematic Review and Meta-Analysis. Canadian Journal of Infectious Diseases and Medical Microbiology. 2022;2022.

2. Wake AD. The acceptance rate toward COVID-19 vaccine in Africa: a systematic review and meta-analysis. Global pediatric health. 2021;8:2333794X211048738.

3. Alemayehu A, Demissie A, Yusuf M, Gemechu Lencha A, Oljira L. Covid-19 Vaccine Acceptance and Determinant Factors among General Public in East Africa: A Systematic Review and Meta-Analysis. Health Services Research and Managerial Epidemiology. 2022;9:23333928221106269.

4. Wake AD. The willingness to receive COVID-19 vaccine and its associated factors:“vaccination refusal could prolong the war of this pandemic”–a systematic review. Risk management and healthcare policy. 2021:2609-23.

5. Mose A, Wasie A, Shitu S, Haile K, Timerga A, Melis T, et al. Determinants of COVID-19 vaccine acceptance in Ethiopia: A systematic review and meta-analysis. PloS one. 2022;17(6):e0269273.

6. Desye B. Prevalence and determinants of COVID-19 vaccine acceptance among healthcare workers: a systematic review. Frontiers in public health. 2022;10.

7. Mengistu DA, Demmu YM, Asefa YA. Global COVID-19 vaccine acceptance rate: Systematic review and meta-analysis. Frontiers in Public Health. 2022;10:1044193.

8. Belay GM, Alemu TG, Techane MA, Wubneh CA, Assimamaw NT, Tamir TT, et al. COVID-19 vaccine acceptance rate and its predictors in Ethiopia: A systematic review and meta-analysis. Human Vaccines & Immunotherapeutics. 2022;18(6):2114699.

9. Yehualashet DE, Seboka BT, Tesfa GA, Mamo TT, Yawo MN, Hailegebreal S. Prevalence and determinants of COVID-19 vaccine hesitancy among the Ethiopian population: a systematic review. Risk Management and Healthcare Policy. 2022:1433-45.

10. Yasmin F, Najeeb H, Moeed A, Naeem U, Asghar MS, Chughtai NU, et al. COVID-19 vaccine hesitancy in the United States: a systematic review. Frontiers in public health. 2021;9:770985.

11. Gudayu TW, Mengistie HT. COVID-19 vaccine acceptance in sub-Saharan African countries: A systematic review and meta-analysis. Heliyon. 2023:e13037.

12. Akem Dimala C, Kadia BM, Nguyen H, Donato A. Community and Provider Acceptability of the COVID-19 Vaccine: A Systematic Review and Meta-analysis. Advances in Clinical Medical Research and Healthcare Delivery. 2021;1(3):1.

13. Norhayati MN, Che Yusof R, Azman YM. Systematic review and meta-analysis of COVID-19 vaccination acceptance. Frontiers in medicine. 2022;8:3091.

14. Jarrett C, Wilson R, O’Leary M, Eckersberger E, Larson HJ. Strategies for addressing vaccine hesitancy–A systematic review. Vaccine. 2015;33(34):4180-90.

15. Wang Q, Yang L, Jin H, Lin L. Vaccination against COVID-19: A systematic review and meta-analysis of acceptability and its predictors. Preventive medicine. 2021;150:106694.

16. Kukreti S, Rifai A, Padmalatha S, Lin C-Y, Yu T, Ko W-C, et al. Willingness to obtain COVID-19 vaccination in general population: a systematic review and meta-analysis. J Glob Health. 2022;12(6):50.

17. Moltot T, Lemma T, Silesh M, Sisay M, Shewangizaw A, Getaneh T, et al. COVID-19 vaccine acceptance among health care professionals in Ethiopia: A systematic review and meta-analysis. Human Vaccines & Immunotherapeutics. 2023;19(1):2188854.

18. Nindrea RD, Usman E, Katar Y, Sari NP. Acceptance of COVID-19 vaccination and correlated variables among global populations: A systematic review and meta-analysis. Clinical epidemiology and global health. 2021;12:100899.

19. Olu-Abiodun O, Abiodun O, Okafor N. COVID-19 vaccination in Nigeria: A rapid review of vaccine acceptance rate and the associated factors. PloS one. 2022;17(5):e0267691.

20. Mahmud S, Mohsin M, Hossain S, Islam MM, Muyeed A. The acceptance of COVID-19 vaccine: a global rapid systematic review and meta-analysis. Available at SSRN 3855987. 2021.

21. Nehal KR, Steendam LM, Campos Ponce M, van der Hoeven M, Smit GSA. Worldwide vaccination willingness for COVID-19: a systematic review and meta-analysis. Vaccines. 2021;9(10):1071.

22. Shakeel CS, Mujeeb AA, Mirza MS, Chaudhry B, Khan SJ. Global COVID-19 vaccine acceptance: a systematic review of associated social and behavioral factors. Vaccines. 2022;10(1):110.
